# Safety and Efficacy of Sodium-Glucose Transport Protein 2 Inhibitors and Glucagon-like Peptide-1 Receptor Agonists in Diabetic Kidney Transplant Recipients: Synthesis of Evidence

**DOI:** 10.3390/jcm13206181

**Published:** 2024-10-17

**Authors:** Ioannis Bellos, Pagona Lagiou, Vassiliki Benetou, Smaragdi Marinaki

**Affiliations:** 1 Department of Hygiene, Epidemiology and Medical Statistics, School of Medicine, National and Kapodistrian University of Athens, 75, Mikras Asias Str., 115 27 Athens, Greecevbenetou@med.uoa.gr (V.B.); 2 Department of Nephrology and Renal Transplantation, Laiko General Hospital, School of Medicine, National and Kapodistrian University of Athens, 115 27 Athens, Greece; smaragdimarinaki@yahoo.com

**Keywords:** sglt2, glp1, antidiabetic, diabetes mellitus, kidney transplantation, meta-analysis

## Abstract

**Background:** This systematic review and meta-analysis aimed to evaluate the efficacy and safety of novel antidiabetics, namely, sodium-glucose transport protein 2 inhibitors (SGLT2-i) and glucagon-like peptide-1 receptor agonists (GLP1-RA), in diabetic kidney transplant recipients. **Methods:** Medline, Scopus, Web of Science, CENTRAL, and Clinicaltrials.gov were systematically searched from inception until 25 August 2024. Pooled estimates were obtained by applying random-effects models. **Results:** Overall, 18 studies (17 observational studies and one randomized controlled trial) were included. GLP1-RA were administered to 270 and SGLT2-i to 1003 patients. After GLP1-RA therapy, patients presented significantly lower glycated hemoglobin [mean difference (MD): −0.61%; 95% confidence interval (CI): −0.99; −0.23] and body weight (MD: −3.32 kg; 95% CI: −5.04; −1.59) but a similar estimated glomerular filtration rate (eGFR) and systolic blood pressure. After SGLT2-i therapy, patients had significantly lower glycated hemoglobin (MD: −0.40%, 95% CI: −0.57; −0.23) and body weight (MD: −2.21 kg, 95% CI: −2.74; −1.67), while no difference was noted in eGFR or systolic blood pressure. Preliminary data have shown an association between SGLT2-i use and a reduced risk of cardiovascular events, graft loss, and mortality. Evidence regarding the association between GLP1-RA and SGLT2-i and proteinuria was mixed. No significant effects on calcineurin inhibitor levels were observed. The risk of urinary tract infections was similar among patients treated with SGLT2-i or placebo (odds ratio: 0.84, 95% CI: 0.43; 1.64). **Conclusions:** Observational data suggest that GLP1-RA and SGLT2-i administration in diabetic kidney transplant recipients may be associated with better glycemic control and reduced body weight, presenting an acceptable safety profile.

## 1. Introduction

Cardiovascular disease constitutes a major source of morbidity in kidney transplant recipients and remains the leading cause of mortality with a functioning graft [1]. The pathophysiology of cardiovascular disease after kidney transplantation is multifactorial as it is mediated by the interplay of both traditional and transplant-specific risk factors [2]. In particular, established modifiable cardiovascular risk factors including hypertension, pre-existing or post-transplant diabetes mellitus, and dyslipidemia present high incidence among kidney transplant recipients and are amplified by the adverse metabolic effects of immunosuppressive drugs, low-grade inflammation, and baseline chronic kidney disease [3]. As a result, kidney transplant recipients are at increased risk of progressive atherosclerosis and vascular calcification [4] leading to coronary artery disease, valvular heart disease, pulmonary hypertension, and heart failure [5].

Sodium-glucose co-transporter 2 inhibitors (SGLT2-i) have shown significant cardio-renoprotective effects in chronic kidney disease patients, with or without diabetes mellitus. Specifically, in the CREDENCE trial [6], canagliflozin effectively reduced the risk of both kidney failure and cardiovascular events among patients with albuminuric diabetic kidney disease. In the DAPA-CKD trial [7], dapagliflozin was demonstrated to protect from kidney disease progression and reduce the risk of the composite endpoint of cardiovascular death and hospitalization for heart failure, regardless of the presence of diabetes. In addition, the EMPA-KIDNEY suggested that empagliflozin was able to slow the slope of the estimated glomerular filtration rate (eGFR) decline and decrease the risk of death due to cardiovascular causes, irrespective of chronic kidney disease stage and baseline albuminuria [8,9].

Glucagon-like peptide-1 receptor agonists (GLP1-RA) have also been proposed to reduce cardiovascular risk and improve survival in patients with type 2 diabetes mellitus [10]. Concerning the chronic kidney disease population, secondary analyses of the SUSTAIN 6 and LEADER trials have suggested that semaglutide and liraglutide could lower the degree of albuminuria and decrease the rate of eGFR decline [11]. The renoprotecive effects of GLP1-RA were recently confirmed by the FLOW trial, indicating that among patients with type 2 diabetes and chronic kidney disease, semaglutide therapy was associated with a significantly reduced risk of kidney disease progression, cardiovascular events, and overall mortality [12].

Despite the benefits of SGLT2-i and GLP1-RA in chronic kidney disease, the existing data regarding their efficacy in the kidney transplant population remain sparse, while concerns about potential adverse events may limit their prescription. The present study aims to systematically accumulate the current literature knowledge in the field, in order to evaluate whether treatment of kidney transplant recipients with SGLT2-i and GLP1-RA is associated with improved metabolic and kidney outcomes, as well as to assess the risk of toxicity and drug interactions.

## 2. Materials and Methods

### 2.1. Study Design

This study was designed and reported following the PRISMA (Preferred Reporting Items for Systematic reviews and Meta-Analyses) guidelines [13]. The pre-registered protocol of this systematic review is publicly available (dx.doi.org/10.17504/protocols.io.e6nvw1eq9lmk/v1). No ethical approval was necessary since already published data were exclusively used.

### 2.2. Eligibility Criteria

The population of this study consisted exclusively of kidney transplant recipients, with or without diabetes mellitus. The intervention of interest was the administration of any SGLT2-i or GLP1-RA. The comparator was planned to be placebo or standard care, but one-arm studies were also held as potentially eligible. The efficacy outcomes of interest included all-cause mortality, allograft failure, cardiovascular events (including nonfatal myocardial infarction, stroke, and hospitalization for heart failure), glycated hemoglobin (HbA1c), body weight, eGFR, proteinuria, and systolic blood pressure following treatment with SGLT2-i or GLP1-RA. The safety endpoints included the potential interaction with calcineurin inhibitor (cyclosporine and/or tacrolimus) blood levels, the occurrence of any serious adverse effects, the incidence of urinary tract infections, and the rate of drug discontinuation. Randomized controlled trials (RCTs) and observational (cohort and case-control) studies were potentially eligible. Cross-sectional and descriptive studies, case series (<10 patients), review articles, conference abstracts, and in vitro studies were excluded from the present review.

### 2.3. Literature Search

The following databases were primarily searched in a systematic fashion: Medline (accessed via PubMed), Scopus, Web of Science, CENTRAL (Cochrane Central Register of Controlled Trials), and Clinicaltrials.gov. In addition, Google Scholar and the reference lists of the included studies were also searched to screen for potential additional studies. The literature search was performed from the inception of each database to 25 August 2024. The search was based on a combination of keywords with MeSH (Medical Subject Heading) terms. The main search algorithm was constructed as follows: “((“Glucagon-Like Peptide-1 Receptor Agonists” [Mesh] OR “Glucagon-Like Peptide 1” [Mesh] OR GLP-1 OR “Glucagon-Like Peptide-1 agonist*” OR exenatide OR liraglutide OR albiglutide OR dulaglutide OR lixisenatide OR semaglutide OR tirzepatide) OR (“Sodium-Glucose Transporter 2 Inhibitors” [Mesh] OR “sodium-glucose cotransporter-2 inhibitor*” OR “sglt2 inhibitor*” OR “sglt-2 inhibitor*” OR gliflozin OR canagliflozin OR bexagliflozin OR dapagliflozin OR empagliflozin OR ertugliflozin OR ipragliflozin OR luseogliflozin OR remogliflozin OR sergliflozin OR sotagliflozin OR tofogliflozin OR henagliflozin)) AND (“Kidney Transplantation” [Mesh] OR “kidney transplant*” OR “renal transplant*”)”. No date or language restrictions were applied.

### 2.4. Study Selection

The process of study selection followed 3 consecutive steps. Firstly, the titles and abstracts of all electronic records were screened to assess for potential eligibility. Subsequently, all articles that were considered to fulfill the eligibility criteria of this systematic review were retrieved in full-text form. Then, any study that did not report the outcomes of interest or met any of the exclusion criteria was not included in this review. The inclusion of studies was decided by two authors, resolving any disagreements through their consensus.

### 2.5. Data Collection

The following data were extracted from the included studies using pre-piloted forms: year of publication, study design, country, sample size, investigated drugs, duration of follow-up (in months), percentage of males and patients with diabetes mellitus, median age (in years), weight (in kg), body mass index (in kg/m^2^), and eGFR (in mL/min/1.73 m^2^), as well as the necessary information for the endpoints of interest. Data were extracted independently by two authors, and any discrepancies were resolved through the consensus of all authors.

### 2.6. Risk of Bias Assessment

The methodological quality of RCTs was appraised using the RoB-2 tool [14], assigning low risk, some concerns, or high risk of bias in the domains of randomization, deviations from intended interventions, missing outcome data, outcome measurement, and selection of the reported results. The ROBINS-I tool [15] was applied to evaluate the quality of observational studies. Specifically, the following domains were taken into account: confounding, selection of participants, classification of interventions, departures from intended interventions, missing data, measurement of outcomes, and selection of the reported results. Two authors independently assessed the risk of bias in all included studies; any potential discrepancies were resolved after discussion with all authors.

### 2.7. Statistical Analysis

The statistical analysis was performed in R-4.4.0 (package “*metafor*” [15]). Statistical significance was defined by a two-sided *p*-value ≤ 0.05, setting the confidence intervals (CIs) at 95%. Pool estimates of mean difference (MD) were obtained by fitting random-effects models, using the maximum likelihood method for the estimation of between-study variance. Meta-analysis of continuous aggregate data measured before and after treatment with SGLT2-i was conducted using the web-based MA-cont:pre/post effect size tool [16]. Meta-analysis of proportions was performed using the Freeman–Tukey transformation [17]. Inter-study heterogeneity was quantified by calculating the inconsistency index (*I*^2^) with *I*^2^ values > 50% indicating substantial heterogeneity [18]. The 95% prediction intervals were estimated to assess the effects to be expected by future studies in the field [18]. Trim–fill funnel plots were constructed, and new estimates were obtained after imputing potentially missing studies [19]. The asymmetry of funnel plots was statistically tested using Egger’s regression test only in the case of 10 or more studies per outcome [20]. Meta-regression analysis was performed based on the following parameters: study design, location, follow-up duration, and risk of bias. The endpoints of urinary protein excretion and calcineurin inhibitor levels were analyzed only qualitatively because of the presence of skewed distributions and heterogeneity in evaluation across studies. In addition, no pooled drug discontinuation rates were obtained because of the expected inter-study heterogeneity regarding drug discontinuation criteria.

### 2.8. Certainty of Evidence

The Grading of Recommendations, Assessment, Development, and Evaluations (GRADE) approach [21] was followed to critically assess the certainty of the existing evidence, evaluating the following domains: study limitations, consistency, directness, imprecision, and publication bias. The GRADE assessment was performed by two authors independently, resolving potential disagreements through consensus.

## 3. Results

### 3.1. Study Selection

**Figure 1:**
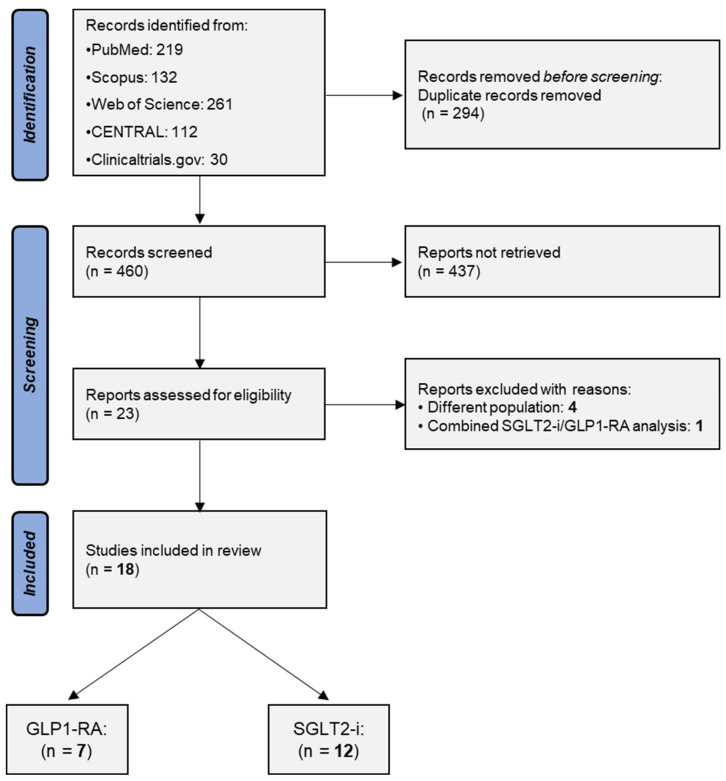
Search plot diagram.

The process of study selection is illustrated in a PRISMA flowchart (Figure 1). Overall, 754 electronic records were identified through the database search. After the removal of duplicate records, the title and abstracts of 460 studies were screened, and 23 of them were retrieved as full texts. Subsequently, five studies were excluded as four of them [22,23,24,25] did not exclusively include kidney transplant recipients, while one study analyzed patients treated with GLP1-RA and SGLT2-i together [26]. As a result, 18 studies [27,28,29,30,31,32,33,34,35,36,37,38,39,40,41,42,43,44] were included in the present review.

### 3.2. Included Studies

The methodological characteristics of the included studies are presented in Table 1. Eight studies were conducted in Asia, six in Europe, and four in the USA. Fourteen studies adopted a retrospective cohort design, three were prospective cohort studies, and one study was a randomized controlled trial. GLP1-RA were administered to 270 and SGLT2-i to 1003 kidney transplant recipients. A control group was included in eight studies (994 participants). The follow-up period ranged from 4 to 72 months. The median percentage of male patients was 66%. The median age of patients ranged from 51.3 to 66 years, while the median eGFR ranged from 38.6 to 73.5 mL/min/1.73 m^2^. The vast majority of patients had pre-existing or post-transplant diabetes mellitus, and only four participants had no history of diabetes. According to the RoB-2 tool, the included randomized controlled trial was evaluated to be at low risk of bias. The ROBINS-I tool indicated a low risk of bias in 1 study, a moderate risk in 11 studies, and a serious risk of bias in 5 studies (Table S1). Concerns of bias were mainly assigned in the domains of confounding and the selection of participants because of differences in baseline characteristics, lack of covariate adjustment, and inadequate description of control group selection. In addition, treating SGLT2-i and GLP1-RA as baseline and not time-dependent variables may have introduced immortal-time bias.

### 3.3. Glucagon-like Peptide-1 Receptor Agonists

The meta-analyses of GLP1-RA effects were based on single-arm studies. The outcomes of quantitative pooling, heterogeneity assessment, and GRADE evaluation are presented in Table 2. The forest plots are illustrated in Figures S1–S4 and the funnel plots in Figures S9–S12.

#### 3.3.1. HbA1c

The change in HbA1c before and after GLP1-RA therapy was examined in six studies [29,30,31,32,34,42]. The pooling of studies suggested that HbA1c was significantly lower following GLP1-RA administration (MD: −0.61%; 95% CI: −0.99 to −0.23) (Figure S1). Statistical heterogeneity was moderate to high (*I*^2^: 57%), while the 95% prediction intervals remained statistically significant, ranging from −1.37 to 0.15 (Table 2). The trim–fill method recognized three potentially missing studies (new MD: −0.25; −0.70 to 0.19) (Figure S9). The meta-regression analysis showed that the outcome may have been influenced by the duration of follow-up, with studies with longer follow-up periods reporting more pronounced effects (*β*: −0.07, *p*-value: 0.008) (Table 3). The certainty of evidence was evaluated as low, downgrading for study limitations and inconsistency.

#### 3.3.2. Body Weight

The body weight change before and after GLP1-RA treatment was assessed in five studies [29,30,31,32,34]. The meta-analysis indicated that body weight was significantly lower after GLP1-RA therapy (MD: −3.32 kg; 95% CI: −5.04 to −1.59) (Figure S2). Statistical heterogeneity was low (*I*^2^: 10.0%), and the 95% prediction intervals ranged from −5.51 to −1.12. The trim–fill method imputed two missing studies (new MD: −3.03; 95% CI: −4.45 to −1.61) (Figure S10). The meta-regression analysis suggested no significant effects of study location, follow-up duration, or risk of bias (Table 3). The certainty of evidence was appraised as moderate because of concerns about study limitations.

**Table 3:** Outcomes of the meta-regression analysis.

Covariate	HbA1c	Weight	eGFR	Systolic Blood Pressure
**GLP1-RA**				
Study location	0.634	0.368	0.701	NA
Study design	NA	NA	NA	NA
Follow-up	**0.008**	0.126	0.265	NA
Risk of bias	0.945	0.511	0.605	NA
**SGLT2-i**				
Study location	0.423	0.807	0.247	**0.035**
Study design	0.273	0.823	0.666	0.969
Follow-up	**0.050**	0.572	0.868	0.518
Risk of bias	0.080	0.909	0.494	**0.035**

Data represent *p*-values. Bold text indicates statistical significance (*p*-value ≤ 0.05). SGLT2-i: sodium-glucose cotransporter-2 inhibitors; GLP1-RA: glucagon-like peptide-1 receptor agonists; HbA1c: glycated hemoglobin; eGFR: estimated glomerular filtration rate; NA: not applicable.

#### 3.3.3. eGFR

The change in eGFR before and after GLP1-RA treatment was evaluated in four studies [30,31,32,34]. The pooling of studies indicated that eGFR did not significantly differ following GLP1-RA administration (MD: 2.01 mL/min/1.73 m^2^, 95% CI: −1.18 to 5.20) (Figure S3). No statistical heterogeneity was noted (*I*^2^: 0%), and no missing studies were imputed by the trim–fill method (Figure S11). The meta-regression analysis showed no significant effects of study location, follow-up duration, or risk of bias (Table 3). According to the GRADE assessment, the certainty of evidence was judged as low, downgrading for study limitations and imprecision.

#### 3.3.4. Systolic Blood Pressure

The pooling of two studies [32,34] indicated that systolic blood pressure did not significantly change following GLP1-RA therapy (MD: −6.31 mmHg, 95% CI: −13.80 to 1.19) (Figure S4). Statistical heterogeneity was low (*I*^2^: 4.1%), and the 95% prediction intervals ranged from −14.12 to 1.51. The certainty of evidence was evaluated as low because of concerns about study limitations and imprecision.

#### 3.3.5. Urinary Protein Excretion

The change in urinary protein excretion following GLP1-RA therapy was examined in four studies (Table S2). Vigara et al. [32] suggested that the urinary albumin-to-creatinine ratio was significantly lower among 26 kidney transplant recipients who completed a 12-month follow-up after GLP1-RA treatment (108.1 vs. 59.6 mg/g, *p*-value: 0.021). In addition, Mahmoud et al. [42] showed a significant reduction in the urinary albumin-to-creatinine ratio at the end of follow-up among patients treated with GLP1-RA compared with the control group. However, no significant difference in urinary protein or albumin excretion following GLP-1RA therapy was observed in three retrospective cohort studies.

#### 3.3.6. Safety

The rate of drug discontinuation ranged from 0% to 29.4% (median: 5.1%) (Table S3). Kukla et al. [31] reported the highest rate, with the main reasons for GLP1-RA discontinuation being gastrointestinal side effects and poor glycemic control, while one case of acute pancreatitis was observed. The rate of urinary tract infections was reported by two studies, ranging from 8.7% to 31.7% (Table S3). In particular, in the study by Mahmoud et al. [42], 13 out of 41 kidney transplant recipients developed 21 episodes of urinary tract infection, although this rate did not significantly differ from the control group. The risk of hypoglycemia was assessed by Kim et al. [29], reporting three cases of mild hypoglycemia (8.1%), without the need for hospitalization. Five studies evaluated the potential influence of GLP1-RA on blood tacrolimus levels, indicating no significant change before and after GLP1-RA treatment. Kim et al. [29] reported that cyclosporine blood levels were lower six months after GLP1-RA initiation, although the statistical significance of the difference was marginal (133.63 vs. 106.00 ng/mL, *p*-value: 0.05). However, Mallik et al. [34] suggested no significant change in blood cyclosporin levels following GLP1-RA therapy (*p*-value: 0.45) (Table S4).

### 3.4. Sodium-Glucose Cotransporter-2 Inhibitors

The meta-analyses of SGLT2-i effects were based on both single-arm and controlled studies. The outcomes of quantitative pooling, heterogeneity assessment, and GRADE evaluation are presented in Table 2. The forest plots are depicted in Figures S5–S8 and the funnel plots in Figures S13–S16.

#### 3.4.1. HbA1c

The meta-analysis of nine studies [35,36,37,38,39,41,42,43,44] suggested that HbA1c was significantly lower following SGLT2-i therapy (MD: −0.40%, 95% CI: −0.57 to −0.23) (Figure S5). Statistical heterogeneity was low (*I*^2^: 12.2%); thus, the 95% predictive intervals remained statistically significant (−0.65 to −0.15). The trim–fill method imputed two potentially missing studies (new MD: −0.35%, 95% CI: −0.52 to −0.18) (Figure S13). The outcome was significantly affected by the follow-up duration, as studies with longer follow-ups reported a more pronounced HbA1c decrease after SGLT2-i therapy (*β*: −0.05, *p*-value: 0.050) (Table 3). The certainty of evidence was assessed as moderate, downgrading for study limitations. A control group was included in six studies; compared to the control group, SGLT2-i administration was associated with significantly lower HbA1c (MD: −0.85%, 95% CI: −1.52 to −0.18, *I*^2^: 98.7%).

#### 3.4.2. Body Weight

The quantitative pooling of eight studies [35,36,37,38,39,41,43,44] indicated that body weight was significantly lower after than before SGLT2-i treatment (MD: −2.21 kg, 95% CI: −2.74 to −1.67) (Figure S6). No statistical heterogeneity was observed (*I*^2^: 0%), and no missing studies were identified by the trim–fill method (Figure S14). The meta-regression analysis suggested no significant effects of study location, design, follow-up, or risk of bias (Table 3). The certainty of evidence was evaluated as moderate because of concerns about study limitations. Five studies included a control group. The pooling of controlled studies showed that SGLT2-i therapy was associated with significantly lower body weight (MD: −3.81 kg, 95% CI: −5.16 to −2.45, *I*^2^: 91.8%), compared with patients receiving standard care.

#### 3.4.3. eGFR

The change in eGFR before and after SGLT2-i treatment was examined in eight studies [35,36,37,38,39,41,43,44], indicating no significant difference (MD: −1.25 mL/min/1.73 m^2^, 95% CI: −2.83 to 0.34) (Figure S7). Statistical heterogeneity was low (*I*^2^: 13.9%), and the 95% predictive intervals ranged from −3.64 to 1.15. The trim–fill method imputed four missing studies (new MD: −2.04, 95% CI: −3.01 to −1.06) (Figure S15). The meta-regression analysis showed no significant effects of study location, design, follow-up, or risk of bias (Table 3). The certainty of evidence was appraised to be low, downgrading for study limitations and publication bias concerns. A control group was included in five studies, indicating no significant eGFR difference between patients receiving SGTL2-is and standard care (MD: 4.59 mL/min/1.73 m^2^, 95% CI: −2.32 to 11.50, *I*^2^: 99.2%).

#### 3.4.4. Systolic Blood Pressure

The meta-analysis of five studies [36,37,38,41,44] showed no significant difference in systolic blood pressure before and after SGLT2-i treatment (MD: −0.91 mmHg, 95% CI: −5.47 to 3.64) (Figure S8). Moderate to high statistical heterogeneity was noted (*I*^2^: 55.2%), while the 95% predictive intervals ranged from −9.40 to 7.58. No missing studies were identified using the trim–fill method (Figure S16). Study location and risk of bias were recognized as potential sources of inter-study heterogeneity (*p*-value: 0.035) (Table 3). The certainty of evidence was assessed as very low, downgrading for study limitations, imprecision, and inconsistency. A control group was included in four studies, indicating no significant systolic blood pressure difference between patients treated with SGTL2-is or standard care (MD: 2.22 mL/min/1.73 m^2^, 95% CI: −6.61 to 11.06, *I*^2^: 97.6%).

#### 3.4.5. Urinary Protein Excretion

The change in urinary protein excretion following SGLT2-i was assessed in seven studies (Table S3). Demir et al. [35] observed a significant reduction in 24 h protein excretion among 36 SGLT2-I kidney transplant recipients (321 vs. 195 mg, *p*-value: 0.008). Additionally, Mahmoud et al. [42] reported that the urinary albumin-to-creatinine ratio was significantly reduced in patients treated with SGLT2-i compared to the control group. Nonetheless, no significant influence of SGLT2-i on urinary protein excretion was noted in five studies.

#### 3.4.6. Patient and Graft Survival

The endpoints of patient and graft survival were evaluated in one retrospective study [40] including 202 kidney transplant recipients treated with SGLT2-i and a control group of 554 patients receiving standard care. Overall mortality risk did not differ significantly between the two groups [hazard ratio (HR): 0.31, 95% CI: 0.07 to 1.32). On the other hand, treatment with SGLT2-i was associated with a significantly lower risk of death-censored graft failure (HR: 0.30, 95% CI: 0.09 to 0.98) and doubling of serum creatinine (HR: 0.45, 95% CI: 0.23 to 0.88).

#### 3.4.7. Cardiovascular Events

The incidence of major adverse cardiovascular events was evaluated in one retrospective study [40] using propensity-score matching, suggesting that the use of SGLT2-i (127 patients) was associated with a significantly lower risk of cardiovascular events (HR: 0.30, 95% CI: 0.10 to 0.88) compared with the control group (127 patients). In addition, SGTL2-i treatment was also associated with a significantly lower risk of myocardial infarction (HR: 0.04, 95% CI: 0.004 to 0.40), while the risk of hospitalization for heart failure (HR: 0.21, 95% CI: 0.02 to 2.76) and stroke (HR: 1.92, 95% CI: 0.31 to 12.03) did not differ significantly between the two groups.

#### 3.4.8. Safety

The rate of SGLT2-i discontinuation ranged from 0% to 43.6% (median: 7.5%) (Table S4). Lemke et al. [39] reported the highest drug discontinuation rate with the main reason being drug cost, followed by kidney function deterioration and urinary tract infection. In the total sample of patients treated with SGLT2-i, two cases of diabetic ketoacidosis were reported by the included studies. The proportion meta-analysis of 12 studies (1001 participants) indicated that the pooled urinary tract infection rate estimate was 36.0% (95% CI: 31.5 to 40.4, I^2^: 38.1%). The risk of urinary tract infection did not differ significantly among SGTL2-i-treated patients and the control group (six studies, odds ratio: 0.84, 95% CI: 0.43 to 1.64, I^2^: 53.0%). Furthermore, no significant difference in tacrolimus levels before and after SGTL2-i therapy was observed in five studies. Cyclosporin levels were reported in one study [37], although the statistical significance of the difference was not statistically tested (Table S4).

## 4. Discussion

The present systematic review and meta-analysis gathered the available literature data regarding the efficacy and safety of GLP1-RA and SGLT2-i in the kidney transplant population. The existing evidence concerns almost exclusively kidney transplant recipients with pre-existing or post-transplant diabetes mellitus. The pooling of observational studies indicated that the use of GLP1-RA was associated with benefits in terms of glycemic control and body weight reduction, without significant effects on kidney function and blood pressure control. On the other hand, SGTL2-i therapy was associated with significantly lower HbA1c and body weight, while preliminary studies suggested that SGLT2-i may also confer important benefits in reducing cardiovascular risk, as well as improving graft and patient survival.

Landmark RCTs have established the role of GLP1-RA as an effective treatment especially in overweight patients, as their administration is associated with sustained reductions in both body weight and cardiovascular risk, even in the non-diabetic population [45,46]. The cardio-renal beneficial effects of GLP1-RA are based on their pleiotropic action, promoting better glycemic control, body weight reduction, lower insulin resistance, and an improved blood lipid profile [47]. Additionally, GLP1-RA have been proposed to attenuate the release of anti-inflammatory and pro-fibrotic factors, as well as to reduce oxidative stress and enhance endothelial function [48]. Interestingly, GLP1-RA have been suggested to serve as T cell-negative costimulatory molecules and therefore may confer theoretical benefits in terms of allograft rejection risk [49]. It should be also noted that GLP1-RA therapy has been shown to be favorable in patients surviving a severe acute kidney injury episode, as it has been associated with significantly lower long-term risk of cardiovascular events, kidney disease progression, and overall survival [50]. Nonetheless, current evidence regarding hard outcomes in the transplant population remains limited but promising. For example, Dotan et al. [22] suggested that among solid organ (kidney, liver, lung, heart) transplant recipients, GLP1-RA use may be linked to significantly lower risk of cardiovascular events, peripheral vascular disease, and mortality. However, further large-scale prospective studies in the kidney transplant population are needed before conclusions can be safely drawn.

In the general population, the main concerns about GLP1-RA administration include the rare occurrence of serious gastrointestinal adverse events, especially pancreatitis, bowel obstruction, and gastroparesis [51]. The present systematic review suggested that among kidney transplant recipients, mild gastrointestinal adverse effects, such as nausea and vomiting, were the most common complications of GLP1-RA therapy, while only one case (0.4%) of pancreatitis has been reported. The frequency of confirmed hypoglycemia (the mild cases) was lower than expected [52], although the potential underreporting of mild cases may have underestimated its actual incidence. Importantly, no significant influence on blood tacrolimus levels was observed, despite theoretical concerns about reduced absorption due to GLPA1-RA-induced vomiting.

Growing evidence supports the renoprotective effects of SGLT2-i, as their administration has been linked to the long-term preservation of kidney function [53,54]. It has been hypothesized that SGLT2-i may alleviate renal hyperfiltration by normalizing the tubuloglomerular feedback mechanism and restoring adenosine generation [55]. Additionally, SGLT2-i have been shown to reduce albuminuria, improve renal oxygen delivery, and ameliorate glucotoxicity in the proximal tubules, subsequently limiting cellular oxidative stress and the release of pro-fibrotic mediators [56]. Current evidence concerning the influence of SGLT2-i on proteinuria after kidney transplantation is mixed. Although Demir et al. [35] suggested an important 24 h proteinuria reduction following SGLT2-i therapy, this effect was not confirmed by studies including patients with lower baseline urinary protein excretion. Furthermore, limited data exist regarding the potential beneficial effects of SGLT2-i in kidney function after kidney transplantation, as a retrospective study pointed towards a significant reduction in the risk of allograft loss and serum creatinine doubling among SGLT2-i-treated kidney transplant recipients [40]. Nevertheless, further studies are necessary to confirm this finding and shed more light on the exact effects of SGLT2-i treatment on kidney function preservation and long-term graft survival.

SGLT2-i prescription may be mitigated by concerns about glomerular hypoperfusion leading potentially to kidney function deterioration, especially in the setting of hypovolemia [57]. In the present meta-analysis, no significant eGFR difference was observed before and after SGLT2-i administration, although the analysis may have been underpowered and not properly designed to detect an acute eGFR drop. Interestingly, preliminary evidence has suggested that SGLT2-i treatment may correct fluid overload among hyperhydrated kidney transplant recipients, without adversely affecting euvolemic patients [27]. However, large-scale real-world data in the chronic kidney population have pointed towards an increased risk of acute kidney injury even 3 months after SGTL2-i initiation, highlighting the volume depletion risk deriving from natriuresis and glucosuria-induced osmotic diuresis [58]. As a result, patients and healthcare providers need to be vigilant in implementing sick day medication guidance in the presence of early signs of volume depletion or dehydration [59]. Currently, only two cases of euglycemic diabetic ketoacidosis have been reported among SGLT2-i-treated kidney transplant recipients; however, alertness is needed to achieve timely diagnosis by screening for anion gap acidosis and ketonuria, while patients should be educated about the possibility of ketoacidosis even in the case of normal finger-stick glucose measurements. It is important to note that GLP1-RA could also promote volume depletion and contribute to the development of acute kidney injury, mainly because of gastrointestinal losses in the context of adverse effects, such as vomiting and diarrhea [60].

The prescription of SGLT2-i in kidney transplant recipients may be also limited by concerns about a heightened risk of genitourinary infections, deriving from their glucosuric effects. In the context of RCTs, an increased risk of genital infections has been observed in the CANVAS Program [61], the DECLARE-TIMI 58 [62], and the EMPA-REG [63] trials; however, no such safety signal was detected in the DAPA-CKD trial [7]. On the other hand, real-world observational evidence has suggested that SGLT2-i use, compared with dipeptidyl peptidase-4 inhibitors use, may be associated with an even lower risk of hospitalizations due to genitourinary infections among patients with and without chronic kidney disease [58]. In the present meta-analysis, the frequency of urinary tract infections among SGTL2-i users did not differ significantly from the control group, supporting the safety of SGLT2-i in the kidney transplant population.

The present systematic review has several strengths. A comprehensive literature review was conducted, and multiple electronic databases were systematically searched without date restrictions, limiting the risk of article losses. A variety of efficacy and safety outcomes were evaluated, aiming to clarify the role of both GLP1-RA and SGLT2-i in the kidney transplant population. This study updated the outcomes of a previous meta-analysis regarding the effects of GLP1-RA [64], avoiding the inappropriate statistical pooling of studies in case the normality assumption was not met. In addition, the certainty of evidence was critically assessed following the GRADE approach, providing a realistic evaluation of the quality of the existing data in the field. In this context, it should be acknowledged that the current evidence is mainly based on small-scale observational studies at moderate to serious risk of bias, with confounding and various forms of selection bias complicating the safe interpretation of outcomes. More specifically, the reliability of the reported results may be limited by the inadequate adjustment for important covariates in combination with the potential risk of bias in the selection of participants and the lack of a comparator group in single-arm studies. The number of existing studies was small, especially concerning cardiovascular endpoints; thus, further research is needed before safe conclusions can be reached. It should be also noted that the existing evidence concerns almost exclusively kidney transplant recipients with diabetes mellitus; thus, the generalization of outcomes in the non-diabetic population is not feasible.

Based on the established benefits of GLP1-RA and SGLT2-i in chronic kidney disease and the promising data about their efficacy and safety after kidney transplantation, the prescription of these novel anti-diabetic agents is expected to grow among kidney transplant recipients. However, despite the significant rise in SGLT2-i off-label use after kidney transplantation, important disparities have already emerged as less frequent SGLT2-i treatment has been observed among female and Black kidney transplant recipients, as well as those with a lower education status [65]. As a result, these disparities need to be taken into account by policymakers, aiming to render GLP1-RA and SGLT2-i accessible to all patients who may benefit from their administration. Further large-scale studies are also needed to verify the safety of these agents in kidney transplant recipients with and without diabetes mellitus and shed more light on their effectiveness on hard outcomes, especially the risk of kidney disease progression, cardiovascular events, and patient survival. Moreover, given the potential concerns about kidney function deterioration, especially following the initiation of SGLT2-i, future studies should determine the optimal timing for their prescription after kidney transplantation, depending on early graft function and kidney function stability. Importantly, since concerns about urinary tract infection risk are expected to mitigate the administration of SGLT2-i, especially in kidney transplant recipients with recurrent urinary tract infection episodes, high immunosuppression status, or altered urinary tract anatomy, large-scale studies are needed to define the patient subpopulation in which SGTL2-is would provide an optimal cost–benefit ratio.

In conclusion, the present systematic review and meta-analysis suggests that GLP1-RA and SGLT2-i administration in diabetic kidney transplant recipients is associated with better glycemic control and reduced body weight, presenting an acceptable safety profile. As current evidence is mainly observational, well-designed large-scale studies are warranted to elucidate the effects of GLP1-RA and SGLT2-i on hard outcomes after kidney transplantation.

## Figures and Tables

**Table 1:** Methodological characteristics of the included studies.

Study	Country	Sample Size	Design	Investigated Drugs	Maintenance Immunosuppression	Follow-Up (Months)	Male Sex (%)	Age (Years)	Weight (kg)	BMI (kg/m^2^)	Diabetes Mellitus (%)	eGFR (mL/min/1.73 m^2^)
2024; Mahzari [30]	Saudi Arabia	GLP1-RA: 39	RC	Semaglutide	Mycophenolate (100%), tacrolimus (97.4%) cyclosporine (2.6%), steroids (97.4%)	18	74	54	95.7	NR	100 PTDM: 46	73.5
2024; Lim [28]	South Korea	SGLT2-i: 129 Control: 127	RC	Empagliflozin, dapagliflozin	Tacrolimus (87%), cyclosporine (14.2%)	56.3	70.5	54.5	NR	25.3	100 PTDM: 16.9	NR
2024; Schork [27]	Germany	SGLT2-i: 22	PC	Dapagliflozin	NR	6	72.7	61	85.5	27.3	81.8 PTDM: 36.4	38.6
2023; Mahmoud [42]	Kuwait	SGLT2-i: 98 GLP1-RA: 41 Control: 70	RC	Canagliflozin, dulaglutide	Tacrolimus (75.1%), cyclosporine (19.1%)	12	62.2	55.7	NR	31.3	100 PTDM: 41.1	66.3
2023; Mallik [34]	U.K.	GLP1-RA: 23	RC	Dulaglutide, liraglutide	NR	24	65	56.5	NR	NR	100	NR
2023; Fructuoso [36]	Spain	SGLT-2: 323	PC	Empagliflozin, dapagliflozin, canagliflozin, ertugliflozin	Mycophenolate (79.6%), tacrolimus (87.9%), cyclosporine (2.9%), everolimus (9.1%), sirolimus (9.4%), steroids (57.2%)	6	73.7	61.6	81.5	NR	100 PTDM: 60.5	58.4
2023; Demir [35]	Turkey	SGLT2-i: 36 Control: 21	RC	Empagliflozin, dapagliflozin	Mycophenolate, tacrolimus, steroids	12	63.2	51.3	78.5	28.5	100 PTDM: 54.4	72.7
2023; Yeggalam [44]	USA	SGLT2-i: 44 Control: 70	RC	NR	NR	12	63.2	61.5	94.6	31.5	100 PTDM: 46.5	53.9
2022; Vigara [32]	Spain	GLP1-RA: 40	RC	Semaglutide, liraglutide, dulaglutide	Mycopheolate (95%), tacrolimus (100%), everolimus (2.5%), steroids (95%)	12	52.5	62.8	93	35.8	100	46.1
2022; Sato [33]	Japan	GLP1-RA: 73 Control: 73	RC	Liraglutide, dulaglutide, exenatide, lixisenatide	Mycophenolate or everolimus, tacrolimus or cyclosporine, steroids	60	68.1	57.4	NR	24.8	100	45.8
2022; Lemke [39]	USA	SGLT2-i: 39	RC	Canagliflozin, dapagliflozin, empagliflozin	Mycophenolate or azathioprine, tacrolimus or cyclosporine or belatacpt ± steroids	12	74	57	87	30	100 PTDM: 44	62.2
2022; Lim [40]	South Korea	SGLT-2i: 202 Control: 554	RC	Empagliflozin, dapagliflozin	Tacrolimus (81.9%), cyclosporine (19.5%), steroids (97.9%)	72	67.2	52.4	NR	23.9	100 PTDM: 32.8	68.2
2021; Hisadome [38]	Japan	SGLT2-i: 28 Control: 57	RC	Canagliflozin, ipragliflozin, empagliflozin, dapagliflozin, tofogliflozin	Mycophenolate or everolimus, tacrolimus or cyclosporine, steroids	4	30.6	54.8	71.6	23.6	100	49.8
2021; Kim [29]	South Korea	GLP1-RA: 37	RC	Dulaglutide	Tacrolimus (78.4%), cyclosporine (21.6%), steroids (100%)	6	48.6	54.8	72.1	25.7	100	71.7
2020; Kukla [31]	USA	GLP1-RA: 17	RC	Liraglutide, dulaglutide, exenatide	Mycophenolate (88.2%), tacrolimus (94.1%), everolimus (5.9%), steroids (64.7%)	12	65	51.8	101.7	34.1	100 PTDM: 65	53
2020; Song [43]	USA	SGLT2-i: 50	RC	Empagliflozin, canagliflozin, dapagliflozin	Mycophenolate (94%), tacrolimus (90%), steroids (98%)	6	66	57	NR	NR	100 PTDM: 20	66.7
2019; Halden [37]	Norway	SGLT2-i: 22 Control: 22	RCT	Empagliflozin	Mycophenolate (90.9%), tacrolimus (79.5%), cyclosporine (13.6%), everolimus (4.5%), steroids (97.7%)	6	77.3	61	88	28.2	PTDM: 100	62.5
2019; Mahling [41]	Germany	SGLT2-i: 10	PC	Empagliflozin	Mycophenolate (90%), tacrolimus (90%), steroids (20%)	12	80	66	75	NR	100 PTDM: 40	57

SGLT2-i: sodium-glucose cotransporter-2 inhibitors; GLP1-RA: glucagon-like peptide-1 receptor agonists; PC: prospective cohort; RC: retrospective cohort; RCT: randomized controlled trial; PTDM: post-transplant diabetes mellitus; BMI: body mass index; eGFR: estimated glomerular filtration rate; NR: not reported.

**Table 2:** Summary of findings table and certainty of evidence evaluation.

Endpoint	Studies No.	Mean Difference (95% CI)	95% PI	*I* ^2^	Trim-Fill Estimate (95% CI)	GRADE Assessment	
						*Certainty of Evidence*	*Downgrading*
**GLP1-RA**							
HbA1c (%)	6	−0.61 (−0.99; −0.23) *	−1.37; 0.15	57.0%	−0.25 (−0.70; 0.19)	Low	Study limitations, inconsistency
Weight (Kg)	5	−3.32 (−5.04; −1.59) *	−5.51; −1.12 *	10.0%	−3.03 (−4.45; −1.61) *	Moderate	Study limitations
eGFR (mL/min/1.73 m^2^)	4	2.01 (−1.18; 5.20)	−1.18; 5.20	0%	2.01 (−1.18; 5.20)	Low	Study limitations, imprecision
Systolic blood pressure (mmHg)	2	−6.31 (−13.80; 1.19)	−14.12; 1.51	4.1%	NA	Low	Study limitations, imprecision
**SGLT2-i**							
HbA1c (%)	9	−0.40 (−0.57; −0.23) *	−0.65; −0.15 *	12.2%	−0.35 (−0.52; −0.18)	Moderate	Study limitations
Weight (Kg)	8	−2.21 (−2.74; −1.67) *	−2.74; −1.67 *	0%	−2.21 (−2.74; −1.67) *	Moderate	Study limitations
eGFR (mL/min/1.73 m^2^)	8	−1.25 (−2.83; 0.34)	−3.64; 1.15	13.9%	−2.04 (−3.01; −1.06) *	Low	Study limitations, publication bias
Systolic blood pressure (mmHg)	5	−0.91 (−5.47; 3.64)	−9.40; 7.58	55.2%	−0.91 (−5.47; 3.64)	Very low	Study limitations, inconsistency, imprecision

Asterisks denote statistical significance. SGLT2-i: sodium-glucose cotransporter-2 inhibitors; GLP1-RA: glucagon-like peptide-1 receptor agonists; CI: confidence interval; PI: prediction interval; *I*^2^: inconsistency index; GRADE: Grading of Recommendations, Assessment, Development, and Evaluation; HbA1c: glycated hemoglobin; eGFR: estimated glomerular filtration rate; NA: not applicable.

## Data Availability

The extracted data are available in the Supplementary Materials. All data and analyses are available upon request from the corresponding author.

## Supplementary Materials

The following supporting information can be downloaded at https://www.mdpi.com/article/10.3390/jcm13206181/s1: Table S1: Outcomes of the ROBINS-I evaluation of cohort studies; Table S2: Proteinuria before and after GLP1-RA and SGLT2-i therapy; Table S3: Frequency of urinary tract infections and drug discontinuation rate following GLP1-RA and SGLT2-i therapy; Table S4: Calcineurin inhibitor blood levels before and after GLP1-RA and SGLT2-i therapy; Figure S1: Forest plot of HbA1c difference before and after GLP1-RA therapy. *MD: mean difference; CI: confidence interval; df: degrees of freedom*. Figure S2: Forest plot of weight difference before and after GLP1-RA therapy. *MD: mean difference; CI: confidence interval; df: degrees of freedom*. Figure S3: Forest plot of estimated glomerular filtration rate difference before and after GLP1-RA therapy. *MD: mean difference; CI: confidence interval; df: degrees of freedom; eGFR: estimated glomerular filtration rate*. Figure S4: Forest plot of systolic blood pressure difference before and after GLP1-RA therapy. *MD: mean difference; CI: confidence interval; df: degrees of freedom*. Figure S5: Forest plot of HbA1c difference before and after SGLT2-i therapy. *MD: mean difference; CI: confidence interval; df: degrees of freedom*. Figure S6: Forest plot of weight difference before and after SGLT2-i therapy. *MD: mean difference; CI: confidence interval; df: degrees of freedom*. Figure S7: Forest plot of estimated glomerular filtration rate difference before and after SGLT2-i therapy. *MD: mean difference; CI: confidence interval; df: degrees of freedom*. Figure S8: Forest plot of systolic blood pressure difference before and after SGLT2-i therapy. *MD: mean difference; CI: confidence interval; df: degrees of freedom*. Figure S9: Funnel plot of HbA1c difference before and after GLP1-RA therapy. Open circles represent missing studies. Figure S10: Funnel plot of body weight difference before and after GLP1-RA therapy. Open circles represent missing studies; Figure S11: Funnel plot of estimated glomerular filtration rate difference before and after GLP1-RA therapy; Figure S12: Funnel plot of systolic blood pressure difference before and after GLP1-RA therapy; Figure S13: Funnel plot of HbA1c before and after SGLT2-i therapy. Open circles represent missing studies; Figure S14: Funnel plot of body weight before and after SGLT2-i therapy. Open circles represent missing studies; Figure S15: Funnel plot of estimated glomerular filtration rate before and after SGLT2-i therapy. Open circles represent missing studies; Figure S16: Funnel plot of systolic blood pressure before and after SGLT2-i therapy. Open circles represent missing studies.

## References

[1] Ojo A.O., Hanson J.A., Wolfe R.A., Leichtman A.B., Agodoa L.Y., Port F.K. (2000). Long-Term Survival in Renal Transplant Recipients with Graft Function. Kidney Int..

[2] Rangaswami J., Mathew R.O., Parasuraman R., Tantisattamo E., Lubetzky M., Rao S., Yaqub M.S., Birdwell K.A., Bennett W., Dalal P. (2019). Cardiovascular Disease in the Kidney Transplant Recipient: Epidemiology, Diagnosis and Management Strategies. Nephrol. Dial. Transplant..

[3] Aziz F., Jorgenson M., Garg N., Parajuli S., Mohamed M., Raza F., Mandelbrot D., Djamali A., Dhingra R. (2022). New Approaches to Cardiovascular Disease and Its Management in Kidney Transplant Recipients. Transplantation.

[4] Podestà M.A., Cucchiari D., Ciceri P., Messa P., Torregrosa J.V., Cozzolino M. (2022). Cardiovascular Calcifications in Kidney Transplant Recipients. Nephrol. Dial. Transplant..

[5] Chukwu C.A., Rao A., Middleton R., Kalra P.A. (2024). Post-Transplant Cardiovascular Disease in Kidney Transplant Recipients: Incidence, Risk Factors, and Outcomes in the Era of Modern Immunosuppression. J. Clin. Med..

[6] Perkovic V., Jardine M.J., Neal B., Bompoint S., Heerspink H.J.L., Charytan D.M., Edwards R., Agarwal R., Bakris G., Bull S. (2019). Canagliflozin and Renal Outcomes in Type 2 Diabetes and Nephropathy. N. Engl. J. Med..

[7] Heerspink H.J.L., Stefánsson B.V., Correa-Rotter R., Chertow G.M., Greene T., Hou F.-F., Mann J.F.E., McMurray J.J.V., Lindberg M., Rossing P. (2020). Dapagliflozin in Patients with Chronic Kidney Disease. N. Engl. J. Med..

[8] Herrington W.G., Staplin N., Wanner C., Green J.B., Hauske S.J., Emberson J.R., Preiss D., Judge P., Mayne K.J., The EMPA-KIDNEY Collaborative Group (2023). Empagliflozin in Patients with Chronic Kidney Disease. N. Engl. J. Med..

[9] Staplin N., Haynes R., Judge P.K., Wanner C., Green J.B., Emberson J., Preiss D., Mayne K.J., Ng S.Y.A., Sammons E. (2024). Effects of Empagliflozin on Progression of Chronic Kidney Disease: A Prespecified Secondary Analysis from the Empa-Kidney Trial. Lancet Diabetes Endocrinol..

[10] Sattar N., Lee M.M.Y., Kristensen S.L., Branch K.R.H., Del Prato S., Khurmi N.S., Lam C.S.P., Lopes R.D., McMurray J.J.V., Pratley R.E. (2021). Cardiovascular, Mortality, and Kidney Outcomes with GLP-1 Receptor Agonists in Patients with Type 2 Diabetes: A Systematic Review and Meta-Analysis of Randomised Trials. Lancet Diabetes Endocrinol..

[11] Shaman A.M., Bain S.C., Bakris G.L., Buse J.B., Idorn T., Mahaffey K.W., Mann J.F.E., Nauck M.A., Rasmussen S., Rossing P. (2022). Effect of the Glucagon-Like Peptide-1 Receptor Agonists Semaglutide and Liraglutide on Kidney Outcomes in Patients with Type 2 Diabetes: Pooled Analysis of SUSTAIN 6 and LEADER. Circulation.

[12] Perkovic V., Tuttle K.R., Rossing P., Mahaffey K.W., Mann J.F.E., Bakris G., Baeres F.M.M., Idorn T., Bosch-Traberg H., Lausvig N.L. (2024). Effects of Semaglutide on Chronic Kidney Disease in Patients with Type 2 Diabetes. N. Engl. J. Med..

[13] Page M.J., McKenzie J.E., Bossuyt P.M., Boutron I., Hoffmann T.C., Mulrow C.D., Shamseer L., Tetzlaff J.M., Akl E.A., Brennan S.E. (2021). The PRISMA 2020 Statement: An Updated Guideline for Reporting Systematic Reviews. BMJ.

[14] Sterne J.A.C., Savović J., Page M.J., Elbers R.G., Blencowe N.S., Boutron I., Cates C.J., Cheng H.-Y., Corbett M.S., Eldridge S.M. (2019). RoB 2: A Revised Tool for Assessing Risk of Bias in Randomised Trials. BMJ.

[15] Sterne J.A., Hernán M.A., Reeves B.C., Savović J., Berkman N.D., Viswanathan M., Henry D., Altman D.G., Ansari M.T., Boutron I. (2016). ROBINS-I: A Tool for Assessing Risk of Bias in Non-Randomised Studies of Interventions. BMJ.

[16] Papadimitropoulou K., Riley R.D., Dekkers O.M., Stijnen T., le Cessie S. (2022). MA-Cont:Pre/Post Effect Size: An Interactive Tool for the Meta-Analysis of Continuous Outcomes Using R Shiny. Res. Synth. Methods.

[17] Lin L., Xu C. (2020). Arcsine-Based Transformations for Meta-Analysis of Proportions: Pros, Cons, and Alternatives. Health Sci. Rep..

[18] Higgins J.P.T., Thompson S.G. (2002). Quantifying Heterogeneity in a Meta-Analysis. Stat. Med..

[19] Duval S., Tweedie R. (2000). Trim and Fill: A Simple Funnel-Plot-Based Method of Testing and Adjusting for Publication Bias in Meta-Analysis. Biometrics.

[20] Ioannidis J.P.A., Trikalinos T.A. (2007). The Appropriateness of Asymmetry Tests for Publication Bias in Meta-Analyses: A Large Survey. Can. Med. Assoc. J..

[21] Guyatt G., Oxman A.D., Akl E.A., Kunz R., Vist G., Brozek J., Norris S., Falck-Ytter Y., Glasziou P., deBeer H. (2011). GRADE Guidelines: 1. Introduction—GRADE Evidence Profiles and Summary of Findings Tables. J. Clin. Epidemiol..

[22] Dotan I., Rudman Y., Turjeman A., Akirov A., Steinmetz T., Calvarysky B., Cohen T.D. (2024). Glucagon-like Peptide 1 Receptor Agonists and Cardiovascular Outcomes in Solid Organ Transplant Recipients with Diabetes Mellitus. Transplantation.

[23] Singh P., Pesavento T.E., Washburn K., Walsh D., Meng S. (2019). Largest Single-Centre Experience of Dulaglutide for Management of Diabetes Mellitus in Solid Organ Transplant Recipients. Diabetes Obes. Metab..

[24] Thangavelu T., Lyden E., Shivaswamy V. (2020). A Retrospective Study of Glucagon-Like Peptide 1 Receptor Agonists for the Management of Diabetes After Transplantation. Diabetes Ther..

[25] Sweiss H., Hall R., Zeilmann D., Escamilla J., Bhayana S., Patel R., Long C. (2022). Single-Center Evaluation of Safety & Efficacy of Glucagon-Like Peptide-1 Receptor Agonists in Solid Organ Transplantation. Prog. Transplant..

[26] Yugueros González A., Kanter J., Sancho A., Gavela E., Solá E., Ávila A., Pallardó L.M. (2021). Institutional Experience with New Antidiabetic Drugs in Kidney Transplant. Transplant. Proc..

[27] Schork A., Eberbach M.-L., Artunc F., Bohnert B.N., Eisinger F., Heister D.J., Vosseler D., Nadalin S., Birkenfeld A.L., Heyne N. (2024). SGLT2 Inhibitors Correct Fluid Overload in Adult Kidney Transplant Recipients-A Prospective Observational Study. Transpl. Int..

[28] Lim J.H., Kwon S., Seo Y.J., Kim Y.H., Kwon H., Kim Y.S., Lee H., Kim Y.L., Kim C.D., Park S.H. (2024). Cardioprotective Effect of SGLT2 Inhibitor in Diabetic Kidney Transplant Recipients: A Multicenter Propensity Score Matched Study. Kidney Int. Rep..

[29] Kim H.S., Lee J., Jung C.H., Park J.Y., Lee W.J. (2021). Dulaglutide as an Effective Replacement for Prandial Insulin in Kidney Transplant Recipients with Type 2 Diabetes Mellitus: A Retrospective Review. Diabetes Metab. J..

[30] Mahzari M.M., Alluhayyan O.B., Almutairi M.H., Bayounis M.A., Alrayani Y.H., Omair A.A., Alshahrani A.S. (2024). Safety and Efficacy of Semaglutide in Post Kidney Transplant Patients with Type 2 Diabetes or Post-Transplant Diabetes. J. Clin. Transl. Endocrinol..

[31] Kukla A., Hill J., Merzkani M., Bentall A., Lorenz E.C., Park W.D., D’costa M., Kudva Y.C., Stegall M.D., Shah P. (2020). The Use of GLP1R Agonists for the Treatment of Type 2 Diabetes in Kidney Transplant Recipients. Transplant. Direct.

[32] Vigara L.A., Villanego F., Orellana C., Naranjo J., Torrado J., Garcia T., Mazuecos A. (2022). Effectiveness and Safety of Glucagon-like Peptide-1 Receptor Agonist in a Cohort of Kidney Transplant Recipients. Clin. Transplant..

[33] Sato T., Azuma Y., Ozone C., Okazaki M., Takeda A., Okada M., Futamura K., Hiramitsu T., Goto N., Narumi S. (2023). Possible Advantage of Glucagon-Like Peptide 1 Receptor Agonists for Kidney Transplant Recipients with Type 2 Diabetes. J. Clin. Endocrinol. Metab..

[34] Mallik R., Ali O., Casabar M., Mukuba D., Byrne C., McCafferty K., Yaqoob M.M., Chowdhury T.A. (2023). Glucagon-like Peptide-1 Receptor Analogues in Renal Transplant Recipients with Diabetes: Medium Term Follow of Patients from a Single UK Centre. Diabet. Med..

[35] Demir M.E., Özler T.E., Merhametsiz Ö., Sözener U., Uyar M., Ercan Z., Bardak Demir S., Sezer S., Türkmen Sarıyıldız G. (2023). The Results of SGLT-2 Inhibitors Use in Kidney Transplantation: 1-Year Experiences from Two Centers. Int. Urol. Nephrol..

[36] Sánchez Fructuoso A.I., Raba A.B., Deras E.B., Vigara Sánchez L.A., Cecilio R.V.S., Esteve A.F., Vega L.C., Martínez E.G., González Garcia M.E., Coronado P.S. (2023). Sodium-Glucose Cotransporter-2 Inhibitor Therapy in Kidney Transplant Patients with Type 2 or Post-Transplant Diabetes: An Observational Multicentre Study. Clin. Kidney J..

[37] Strøm Halden T.A., Kvitne K.E., Midtvedt K., Rajakumar L., Robertsen I., Brox J., Bollerslev J., Hartmann A., Asberg A., Jenssen T. (2019). Efficacy and Safety of Empagliflozin in Renal Transplant Recipients with Posttransplant Diabetes Mellitus. Diabetes Care.

[38] Hisadome Y., Mei T., Noguchi H., Ohkuma T., Sato Y., Kaku K., Okabe Y., Nakamura M. (2021). Safety and Efficacy of Sodium-Glucose Cotransporter 2 Inhibitors in Kidney Transplant Recipients with Pretransplant Type 2 Diabetes Mellitus: A Retrospective, Single-Center, Inverse Probability of Treatment Weighting Analysis of 85 Transplant Patients. Transplant. Direct.

[39] Lemke A., Brokmeier H.M., Leung S.B., Mara K.C., Mour G.K., Wadei H.M., Hill J.M., Stegall M., Kudva Y.C., Shah P. (2022). Sodium-Glucose Cotransporter 2 Inhibitors for Treatment of Diabetes Mellitus after Kidney Transplantation. Clin. Transplant..

[40] Lim J.H., Kwon S., Jeon Y., Kim Y.H., Kwon H., Kim Y.S., Lee H., Kim Y.L., Kim C.D., Park S.H. (2022). The Efficacy and Safety of SGLT2 Inhibitor in Diabetic Kidney Transplant Recipients. Transplantation.

[41] Mahling M., Schork A., Nadalin S., Fritsche A., Heyne N., Guthoff M. (2019). Sodium-Glucose Cotransporter 2 (SGLT2) Inhibition in Kidney Transplant Recipients with Diabetes Mellitus. Kidney Blood Press. Res..

[42] Mahmoud T., Yagan J., Hasan A., Gheith O.A., Mostafa M., Rida S., El-Serwi N., Shaker M., Khalid M. (2023). Sodium-Glucose Co-Transporter 2 Inhibitors & Glucagon-like Peptide-1 Receptor Agonists, Efficacy & Safety in Diabetic Kidney Transplant Recipients. Clin. Transplant..

[43] Song C.C., Brown A., Winstead R., Yakubu I., Demehin M., Kumar D., Gupta G. (2020). Early Initiation of Sodium-Glucose Linked Transporter Inhibitors (SGLT-2i) and Associated Metabolic and Electrolyte Outcomes in Diabetic Kidney Transplant Recipients. Endocrinol. Diabetes Metab..

[44] Yeggalam A., Liebich J.A., Yu K., Shrestha E., Nadella S., Ahir V., Newman J., Lentine K.L., Caliskan Y., Abu Al Rub F. (2023). Safety and Efficacy of Sodium-Glucose Co-Transporter-2 Inhibitors in Patients with Kidney Transplantation and Diabetes Mellitus. Diabetes Obes. Metab..

[45] Wilding J.P.H., Batterham R.L., Calanna S., Davies M., Van Gaal L.F., Lingvay I., McGowan B.M., Rosenstock J., Tran M.T.D., Wadden T.A. (2021). Once-Weekly Semaglutide in Adults with Overweight or Obesity. N. Engl. J. Med..

[46] Lincoff A.M., Brown-Frandsen K., Colhoun H.M., Deanfield J., Emerson S.S., Esbjerg S., Hardt-Lindberg S., Hovingh G.K., Kahn S.E., Kushner R.F. (2023). Semaglutide and Cardiovascular Outcomes in Obesity without Diabetes. N. Engl. J. Med..

[47] Caruso I., Giorgino F. (2024). Renal Effects of GLP-1 Receptor Agonists and Tirzepatide in Individuals with Type 2 Diabetes: Seeds of a Promising Future. Endocrine.

[48] Kodera R., Shikata K., Kataoka H.U., Takatsuka T., Miyamoto S., Sasaki M., Kajitani N., Nishishita S., Sarai K., Hirota D. (2011). Glucagon-like Peptide-1 Receptor Agonist Ameliorates Renal Injury through Its Anti-Inflammatory Action without Lowering Blood Glucose Level in a Rat Model of Type 1 Diabetes. Diabetologia.

[49] Ben Nasr M., Usuelli V., Dellepiane S., Seelam A.J., Fiorentino T.V., D’Addio F., Fiorina E., Xu C., Xie Y., Balasubramanian H.B. (2024). Glucagon-like Peptide 1 Receptor Is a T Cell-Negative Costimulatory Molecule. Cell Metab..

[50] Pan H.C., Chen J.Y., Chen H.Y., Yeh F.Y., Sun C.Y., Huang T.T.M., Wu V.C. (2024). GLP-1 Receptor Agonists’ Impact on Cardio-Renal Outcomes and Mortality in T2D with Acute Kidney Disease. Nat. Commun..

[51] Sodhi M., Rezaeianzadeh R., Kezouh A., Etminan M. (2023). Risk of Gastrointestinal Adverse Events Associated with Glucagon-Like Peptide-1 Receptor Agonists for Weight Loss. JAMA.

[52] Htike Z.Z., Zaccardi F., Papamargaritis D., Webb D.R., Khunti K., Davies M.J. (2017). Efficacy and Safety of Glucagon-like Peptide-1 Receptor Agonists in Type 2 Diabetes: A Systematic Review and Mixed-Treatment Comparison Analysis. Diabetes Obes. Metab..

[53] Heerspink H.J.L., Jongs N., Chertow G.M., Langkilde A.M., McMurray J.J.V., Correa-Rotter R., Rossing P., Sjöström C.D., Stefansson B.V., Toto R.D. (2021). Effect of Dapagliflozin on the Rate of Decline in Kidney Function in Patients with Chronic Kidney Disease with and without Type 2 Diabetes: A Prespecified Analysis from the DAPA-CKD Trial. Lancet Diabetes Endocrinol..

[54] Wanner C., Heerspink H.J.L., Zinman B., Inzucchi S.E., Koitka-Weber A., Mattheus M., Hantel S., Woerle H.J., Broedl U.C., Von Eynatten M. (2018). Empagliflozin and Kidney Function Decline in Patients with Type 2 Diabetes: A Slope Analysis from the EMPA-REG OUTCOME Trial. J. Am. Soc. Nephrol..

[55] Fonseca-Correa J.I., Correa-Rotter R. (2021). Sodium-Glucose Cotransporter 2 Inhibitors Mechanisms of Action: A Review. Front. Med..

[56] Eleftheriadis T., Pissas G., Tsogka K., Nikolaou E., Liakopoulos V., Stefanidis I. (2020). A Unifying Model of Glucotoxicity in Human Renal Proximal Tubular Epithelial Cells and the Effect of the SGLT2 Inhibitor Dapagliflozin. Int. Urol. Nephrol..

[57] van Bommel E.J.M., Muskiet M.H.A., van Baar M.J.B., Tonneijck L., Smits M.M., Emanuel A.L., Bozovic A., Danser A.H.J., Geurts F., Hoorn E.J. (2020). The Renal Hemodynamic Effects of the SGLT2 Inhibitor Dapagliflozin Are Caused by Post-Glomerular Vasodilatation Rather than Pre-Glomerular Vasoconstriction in Metformin-Treated Patients with Type 2 Diabetes in the Randomized, Double-Blind RED Trial. Kidney Int..

[58] Narasaki Y., Kovesdy C.P., You A.S., Sumida K., Mallisetty Y., Surbhi S., Thomas F., Amin A.N., Streja E., Kalantar-Zadeh K. (2024). Safety of SGLT2 Inhibitors, DPP-4 Inhibitors, and GLP-1 Receptor Agonists in US Veterans with and without Chronic Kidney Disease: A Population-Based Study. Lancet Reg. Health–Am..

[59] Watson K.E., Dhaliwal K., Robertshaw S., Verdin N., Benterud E., Lamont N., Drall K.M., McBrien K., Donald M., Tsuyuki R.T. (2023). Consensus Recommendations for Sick Day Medication Guidance for People with Diabetes, Kidney, or Cardiovascular Disease: A Modified Delphi Process. Am. J. Kidney Dis..

[60] Dong S., Sun C. (2022). Can Glucagon-like Peptide-1 Receptor Agonists Cause Acute Kidney Injury? An Analytical Study Based on Post-Marketing Approval Pharmacovigilance Data. Front. Endocrinol..

[61] Neal B., Perkovic V., Mahaffey K.W., de Zeeuw D., Fulcher G., Erondu N., Shaw W., Law G., Desai M., Matthews D.R. (2017). Canagliflozin and Cardiovascular and Renal Events in Type 2 Diabetes. N. Engl. J. Med..

[62] Wiviott S.D., Raz I., Bonaca M.P., Mosenzon O., Kato E.T., Cahn A., Silverman M.G., Zelniker T.A., Kuder J.F., Murphy S.A. (2019). Dapagliflozin and Cardiovascular Outcomes in Type 2 Diabetes. N. Engl. J. Med..

[63] Zinman B., Wanner C., Lachin J.M., Fitchett D., Bluhmki E., Hantel S., Mattheus M., Devins T., Johansen O.E., Woerle H.J. (2015). Empagliflozin, Cardiovascular Outcomes, and Mortality in Type 2 Diabetes. N. Engl. J. Med..

[64] Krisanapan P., Suppadungsuk S., Sanpawithayakul K., Thongprayoon C., Pattharanitima P., Tangpanithandee S., Mao M.A., Miao J., Cheungpasitporn W. (2024). Safety and Efficacy of Glucagon-like Peptide-1 Receptor Agonists among Kidney Transplant Recipients: A Systematic Review and Meta-Analysis. Clin. Kidney J..

[65] Lentine K.L., Miyata K.N., Lam N.N., Joseph C., McAdams-DeMarco M., Bae S., Chen Y., Caliskan Y., Sarabu N., Dhindsa S. (2024). Sociodemographic Disparities in Sodium-Glucose Cotransporter-2 Inhibitor Use among US Kidney Transplant Recipients: An Observational Study of Real-World Pharmacy Records. Clin. Transplant..

