# Supplementary material for: Safety and Efficacy of Sodium-Glucose Transport Protein 2 Inhibitors and Glucagon-like Peptide-1 Receptor Agonists in Diabetic Kidney Transplant Recipients: Synthesis of Evidence
Source: J Clin Med. 2024 Oct 17;13(20):6181. doi: 10.3390/jcm13206181 (PMC11508237; doi:10.3390/jcm13206181)
Supplement: Supplementary file 1 [file jcm-13-06181-s001.zip › jcm-3238962-supplementary.pdf]

Supplementary Appendix

Contents

Appendix 1: Risk of bias evaluation 2

Appendix 2: Urinary protein excretion 3

Appendix 3: Adverse events 4

Appendix 4: Calcineurin inhibitor levels 5

Appendix 5: Forest plots 6

Appendix 6: Funnel plots 10

Appendix 1: Risk of bias evaluation

Suppl. Table 1. Outcomes of the ROBINS-I evaluation of cohort studies

Study	*Bias due to confounding*	*Bias in selection of participants into the study*	*Bias in classification of interventions*	*Bias due to deviations from intended interventions*	*Bias due to missing data*	*Bias in measurement of outcomes*	*Bias in selection of the reported result*	*Overall bias*
2024; Mahzari	Serious	Moderate	Low	Low	Moderate	Low	Low	Serious
2024; Lim	Low	Moderate	Low	Low	Low	Low	Low	Moderate
2024; Schork	Moderate	Moderate	Low	Low	Low	Low	Low	Moderate
2023; Mahmoud	Serious	Moderate	Low	Low	Low	Low	Low	Serious
2023; Mallik	Moderate	Moderate	Low	Low	Low	Low	Low	Moderate
2023; Fructuoso	Moderate	Low	Low	Low	Low	Low	Low	Moderate
2023; Demir	Serious	Moderate	Low	Low	Low	Low	Low	Serious
2023; Yeggalam	Serious	Moderate	Low	Low	Moderate	Low	Low	Serious
2022; Vigara	Moderate	Moderate	Low	Low	Low	Low	Low	Moderate
2022; Sato	Moderate	Moderate	Low	Low	Moderate	Low	Moderate	Moderate
2022; Lemke	Moderate	Moderate	Low	Low	Low	Low	Low	Moderate
2022; Lim	Low	Moderate	Low	Low	Low	Low	Low	Moderate
2021; Hisadome	Low	Low	Low	Low	Low	Low	Low	Low
2021; Kim	Serious	Moderate	Low	Low	Moderate	Low	Low	Serious
2020; Kukla	Moderate	Moderate	Low	Low	Low	Low	Low	Moderate
2020; Song	Moderate	Moderate	Low	Low	Low	Low	Low	Moderate
2019; Mahling	Moderate	Moderate	Low	Low	Low	Low	Low	Moderate

Appendix 2: Urinary protein excretion

Suppl. Table 2. Proteinuria before and after GLP1-RA and SGLT2-i therapy

Study	Evaluation method	Baseline	End of follow-up	Individual difference	*P-value*
GLP1-RA					
2024; Mahzari	Albumin-to-creatinine ratio in mg/mmol	6.85 [3.55; 54.5]	5.60 [1.70; 40.18]	NR	0.093
2023; Mahmoud	Albumin-to-creatinine ratio in mg/mmol	4.1	4.0	NR	NR
2023; Mallik	Protein-to-creatinine ratio in mg/mmol	NR	NR	−3.14 [−14.18 to 0.76]	0.87
2022; Vigara	Albumin-to-creatinine ratio in mg/g	108.1 [25.6; 12.5]	59.6 [12.5, 88.2]	NR	**0.021**
2020; Kukla	24-h protein excretion in mg	150 [100; 185]	157 [75; 319]	24 [-8.5; 149]	0.1
SGLT2-i					
2024; Schork	Albumin-to-creatinine ratio in mg/g	73 [33; 174]	NR	-9 [-39; 9]	NS
2023; Fructuoso	Albumin-to-creatinine ratio in mg/g	56 [14; 190]	50 [9; 255]	-3.30 [-17.0; 2.80]	0.339
2023; Demir	24-h protein excretion in mg	321 [45; 2565]	195 [51; 1905]	NR	**0.008**
2023; Mahmoud	Albumin-to-creatinine ratio in mg/mmol	9.0	3.0	NR	NR
2023; Yeggalam	Protein-to-creatinine ratio in mg/g	60±20	90±10	NR	0.95
2021; Hisadome	Protein-to-creatinine ratio in mg/g	410±510	400±430	0±50	NS
2019; Halden	24-h protein excretion in mg	100 [60; 150]	100 [80; 140]	10 [-30; 40]	NS

Data presented as mean±standard deviation or median [interquartile range]. Bold text indicated statistical significance.
SGLT2-i: sodium-glucose cotransporter-2 inhibitors; GLP1-RA: glucagon-like peptide-1 receptor agonists; NS: not significant; NR: not reported

Appendix 3: Adverse events

Suppl. Table 3. Frequency of urinary tract infections and drug discontinuation rate following GLP1-RA and SGLT2-i therapy

Study	Urinary tract infection	Drug discontinuation
GLP1-RA		
2024; Mahzari	NR	2/39 (5.1%)
2023; Mallik	2/23 (8.7%)	3/23 (13.0%)
2023; Mahmoud	13/41 (31.7%)	0/41 (0.0%)
2022; Vigara	NR	2/50 (4.0%)
2021; Kim	NR	NR
2020; Kukla	NR	5/17 (29.4%)
SGLT2-i		
2024; Lim	12/127 (9.4%)	18/129 (14.0%)
2024; Schork	2/22 (9.1%)	1/22 (4.5%)
2023; Mahmoud	15/98 (15.3%)	0/98 (0.0%)
2023; Fructuoso	48/323 (14.9%)	34/323 (10.5%)
2023; Demir	6/36 (16.7%)	1/36 (2.8%)
2023; Yeggalam	5/44 (11.4%)	NR
2022; Lemke	6/39 (15.4%)	17/39 (43.6%)
2022; Lim	12/202 (5.9%)	NR
2021; Hisadome	2/28 (7.1%)	0/28 (0.0%)
2020; Song	7/50 (14%)	9/50 (18%)
2019; Halden	3/22 (13.6%)	1/22 (4.5%)
2019; Mahling	2/10 (20%)	2/10 (20%)

SGLT2-i: sodium-glucose cotransporter-2 inhibitors; GLP1-RA: glucagon-like peptide-1 receptor agonists; NR: not reported

Appendix 4: Calcineurin inhibitor levels

Suppl. Table 4. Calcineurin inhibitor blood levels before and after GLP1-RA and SGLT2-i therapy

	**Cyclosporin blood levels (ng/ml)**				**Tacrolimus blood levels (ng/ml)**			
**Study**	***Baseline***	***End of follow-up***	***Individual difference***	***P-value***	***Baseline***	***End of follow-up***	***Individual difference***	***P-value***
GLP1-RA								
2024; Mahzari	NR	NR	NR	NR	7.2±2.2		NR	NS
2023; Mallik	NR	NR	27 [−28; 129]	0.45	NR	NR	−1.3 [−2; 3.8]	0.95
2022; Vigara	NR	NR	NR	NR	6.8±2.1	6.0±1.9	NR	0.255
2021; Kim	133.63±33.46	106.00±23.18	-27.63±32.65	**0.05**	7.63±1.64	7.12±2.66	-0.51±3.08	0.87
2020; Kukla	NR	NR	NR	NR	NR	NR	NR	NS
SGLT2-i								
2023; Fructuoso	NR	NR	NR	NR	7.01 [6.73; 7.29]	6.86 [6.58; 7.15]	-0.15 [-0.44; 0.15]	0.340
2023; Demir	NR	NR	NR	NR	6.73±2.07	6.09±1.54	NR	NS
2022; Lemke	NR	NR	NR	NR	NR	NR	NR	NS
2021; Hisadome	NR	NR	NR	NR	2.6±1.2	2.7±1.3	NR	NS
2019; Halden	94 [80; 105]	88 [79; 97]	-6 [-8; -1]	NR	5.4 [4.6; 6.9]	5.2 [4.5; 6.2]	-0.05 [-1.1; 0.43]	NS

Data presented as mean±standard deviation or median [interquartile range]. Bold text indicated statistical significance.
SGLT2-i: sodium-glucose cotransporter-2 inhibitors; GLP1-RA: glucagon-like peptide-1 receptor agonists; NS: not significant; NR: not reported

Appendix 5: Forest plots

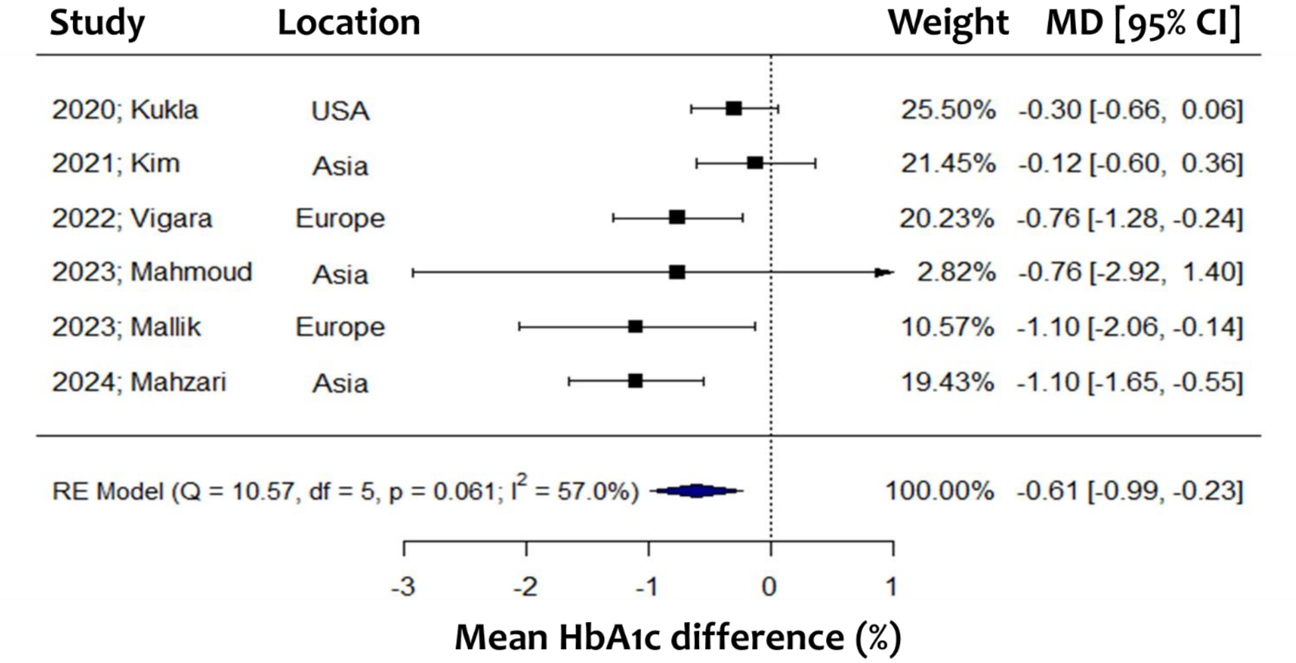

Suppl. Figure 1. Forest plot of HbA1c difference before and after GLP1-RA therapy. *MD: mean difference; CI: confidence intervals; df: degrees of freedom*

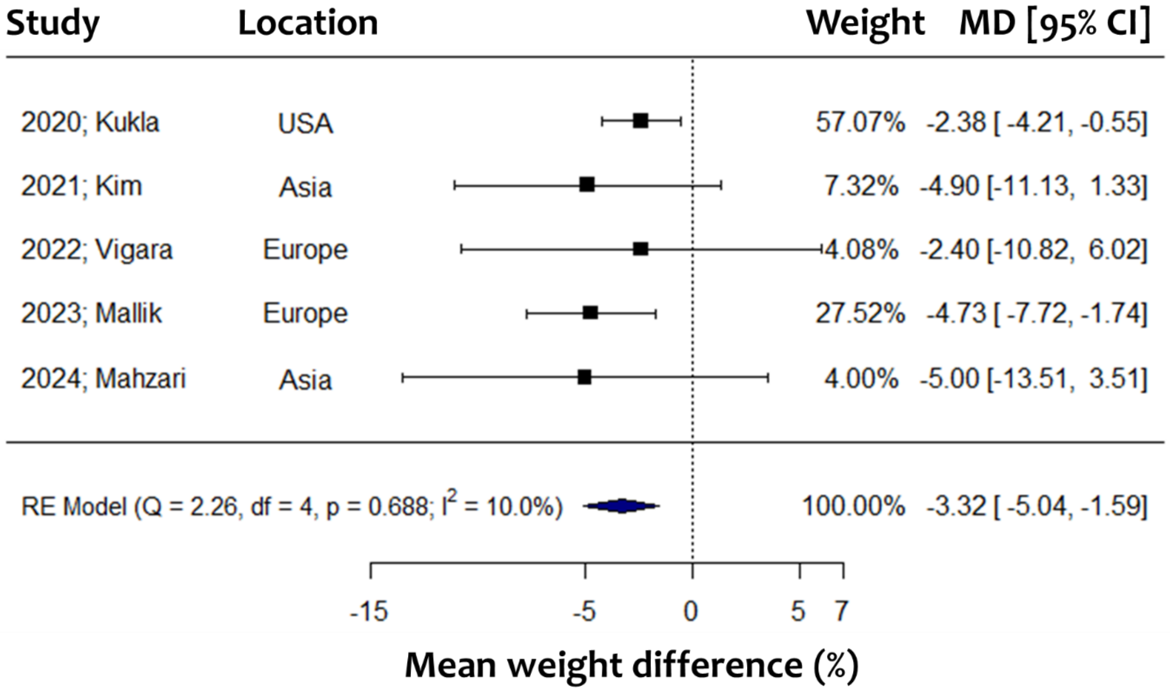

Suppl. Figure 2. Forest plot of weight difference before and after GLP1-RA therapy. *MD: mean difference; CI: confidence intervals; df: degrees of freedom*

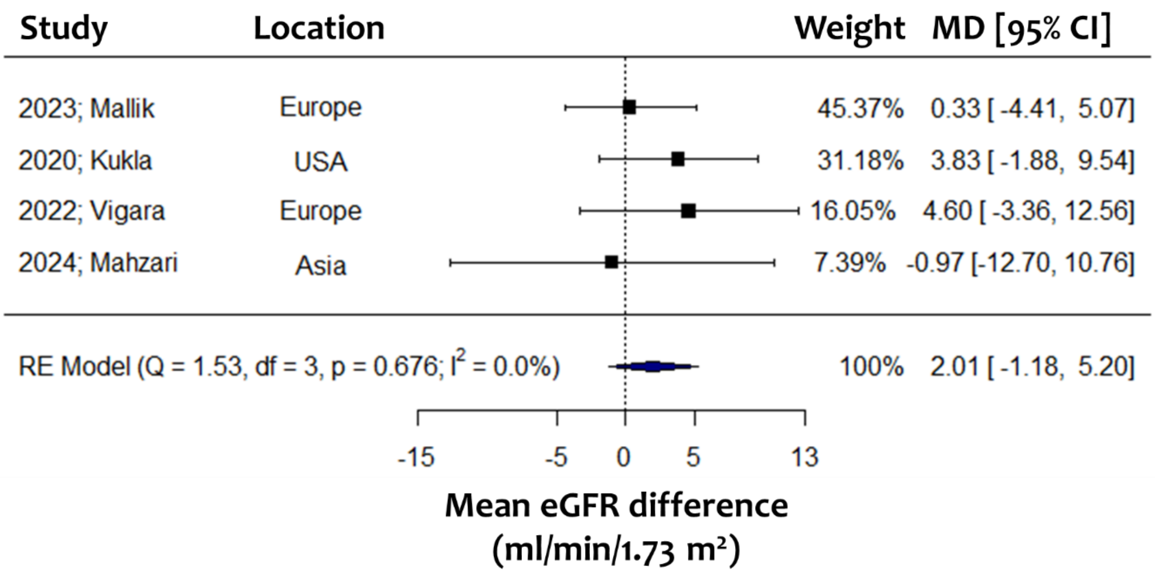

Suppl. Figure 3. Forest plot of estimated glomerular filtration rate difference before and after GLP1-RA therapy. *MD: mean difference; CI: confidence intervals; df: degrees of freedom; eGFR: estimated glomerular filtration rate*

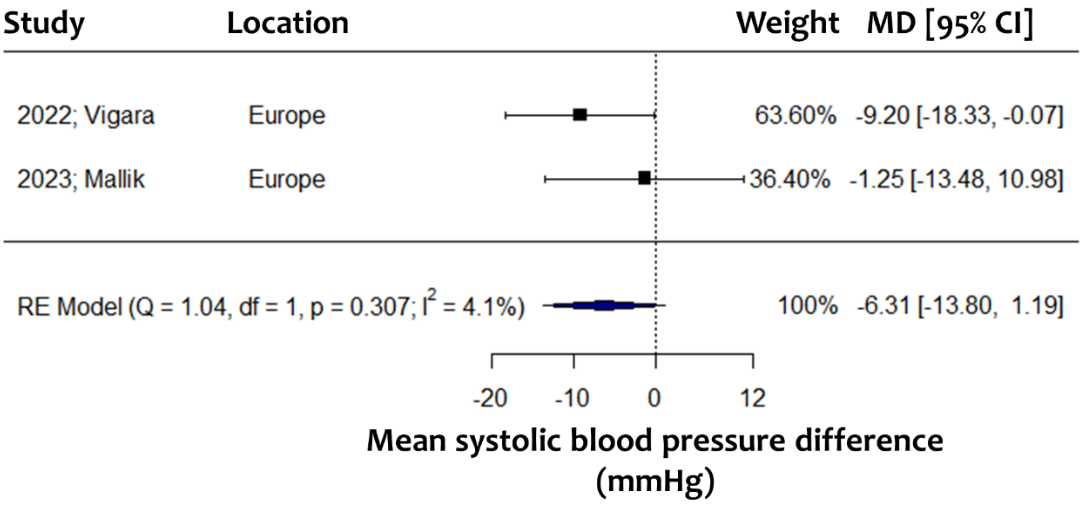

Suppl. Figure 4. Forest plot of systolic blood pressure difference before and after GLP1-RA therapy. *MD: mean difference; CI: confidence intervals; df: degrees of freedom*

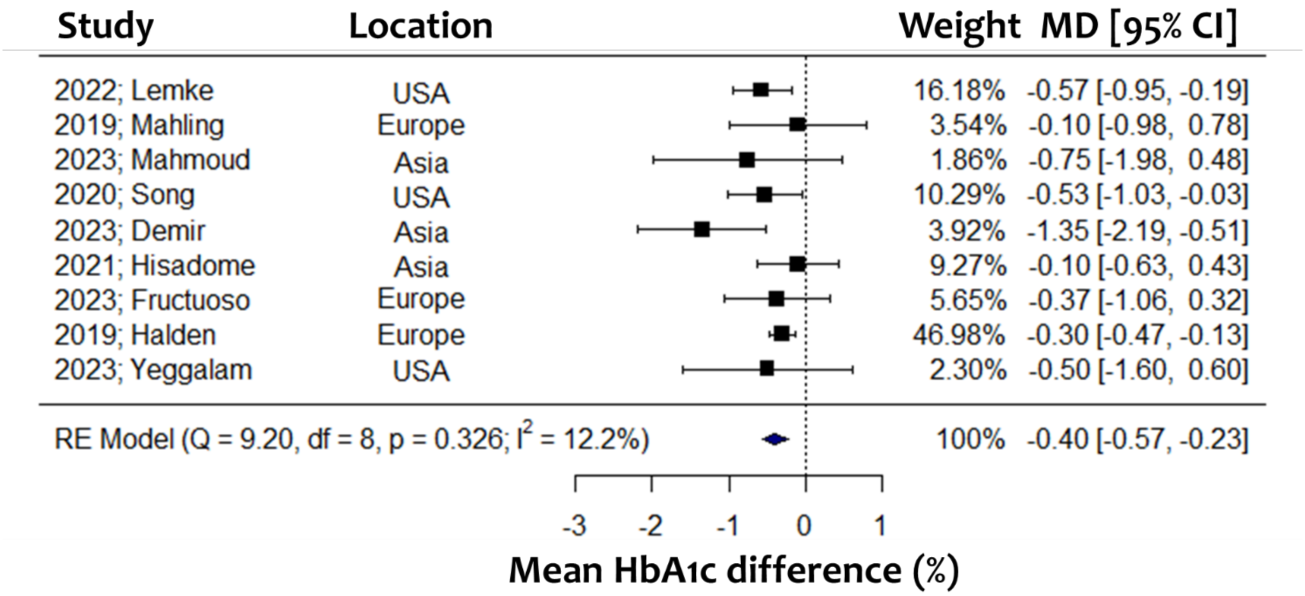

Suppl. Figure 5. Forest plot of HbA1c difference before and after SGLT2-i therapy. *MD: mean difference; CI: confidence intervals; df: degrees of freedom*

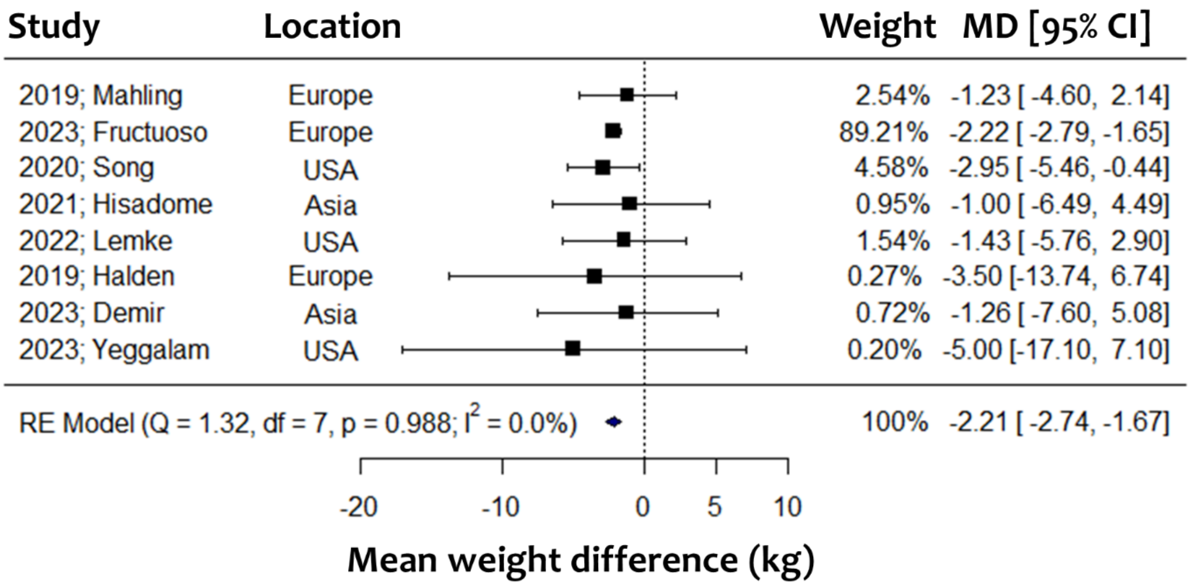

Suppl. Figure 6. Forest plot of weight difference before and after SGLT2-i therapy. *MD: mean difference; CI: confidence intervals; df: degrees of freedom*

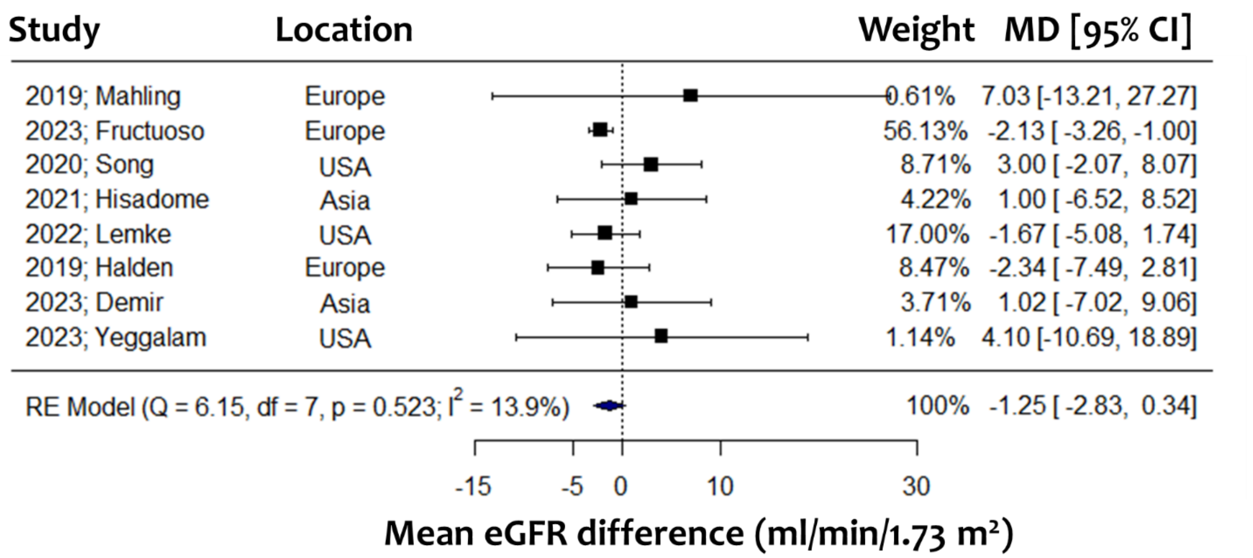

Suppl. Figure 7. Forest plot of estimated glomerular filtration rate difference before and after SGLT2-i therapy. *MD: mean difference; CI: confidence intervals; df: degrees of freedom*

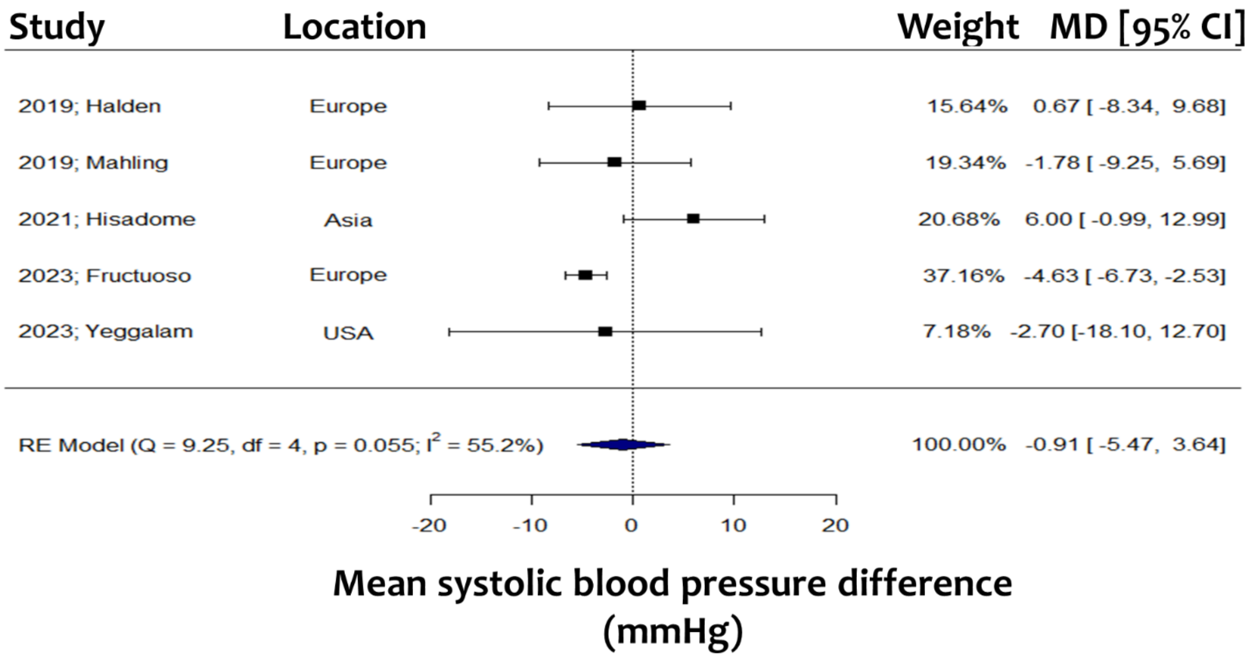

Suppl. Figure 8. Forest plot of systolic blood pressure difference before and after SGLT2-i therapy. *MD: mean difference; CI: confidence intervals; df: degrees of freedom*

Appendix 6: Funnel plots

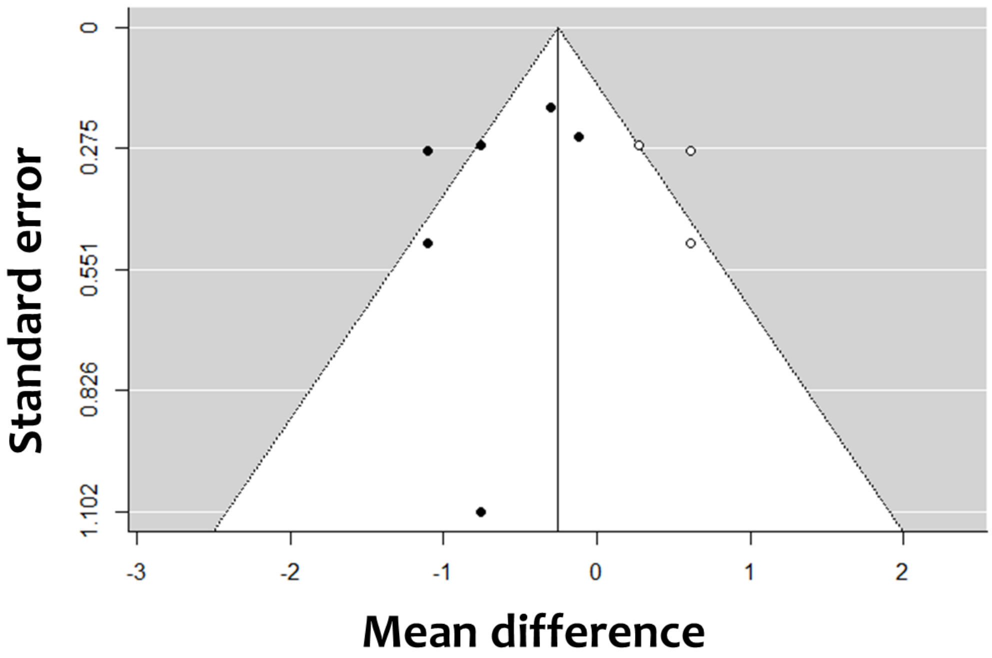

Suppl. Figure 9. Funnel plot of HbA1c difference before and after GLP1-RA therapy. Open circles represent missing studies.

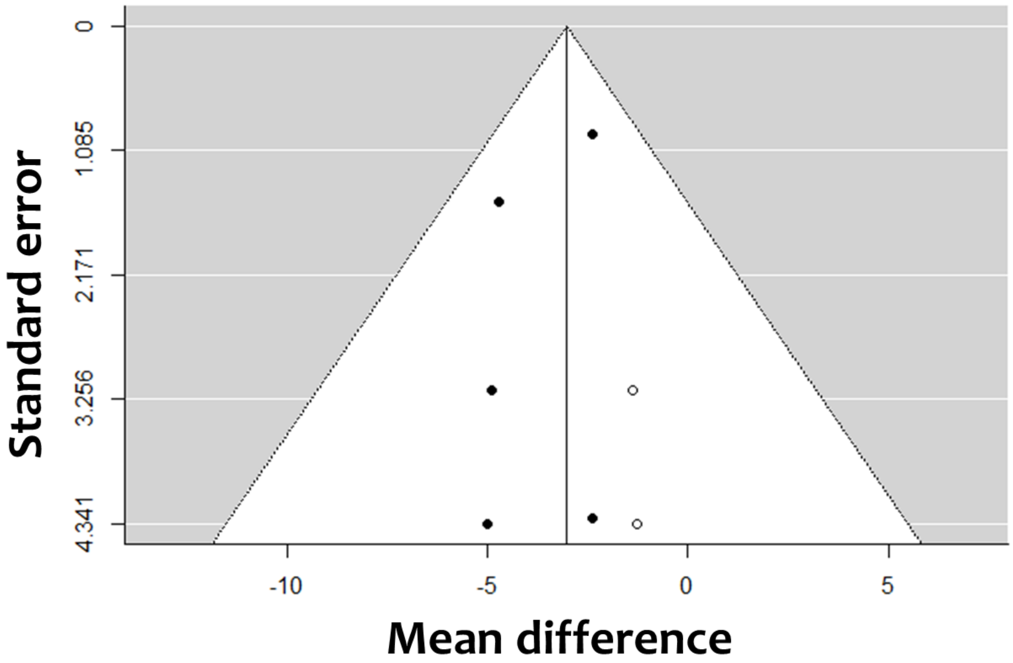

Suppl. Figure 10. Funnel plot of body weight difference before and after GLP1-RA therapy. Open circles represent missing studies.

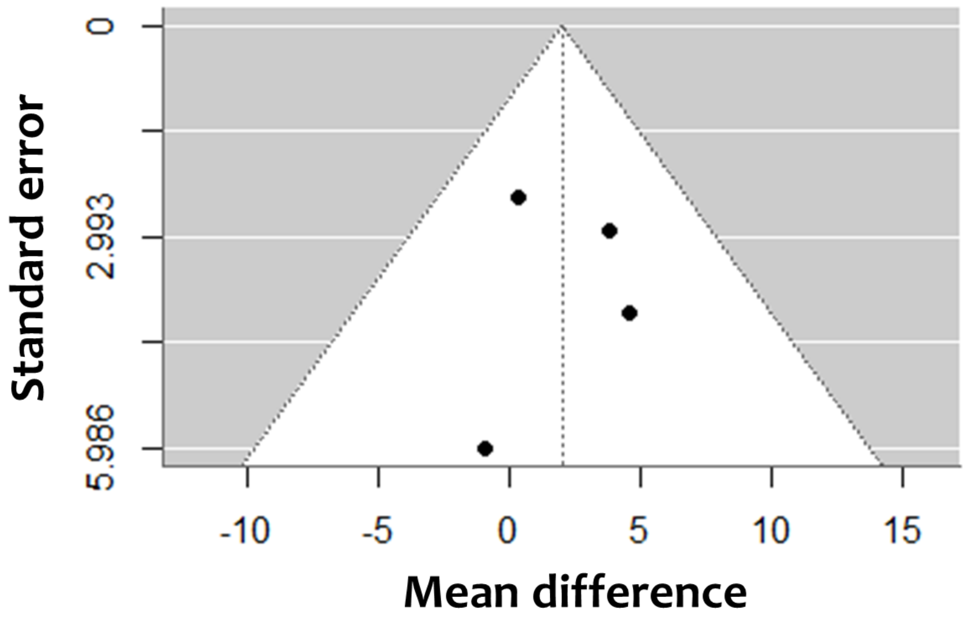

Suppl. Figure 11. Funnel plot of estimated glomerular filtration rate difference before and after GLP1-RA therapy.

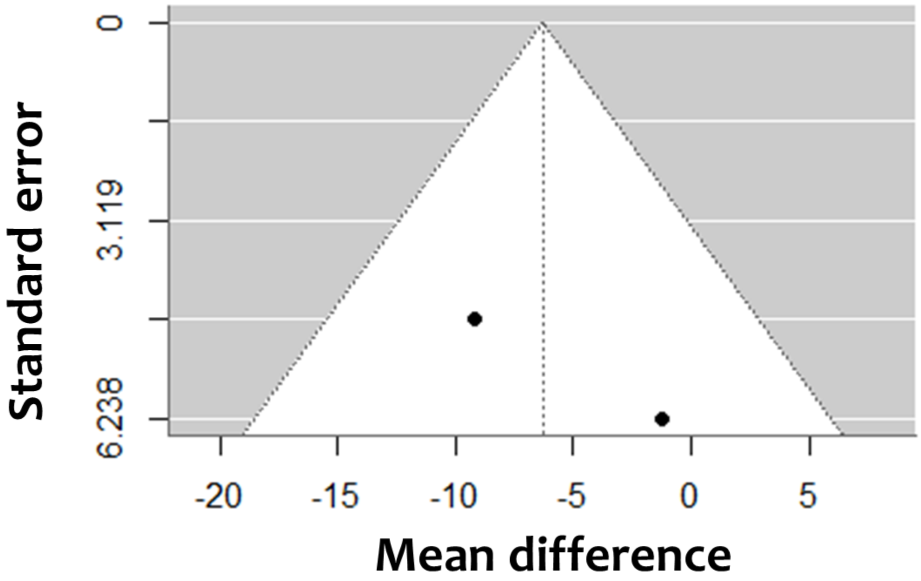

Suppl. Figure 12. Funnel plot of systolic blood pressure difference before and after GLP1-RA therapy.

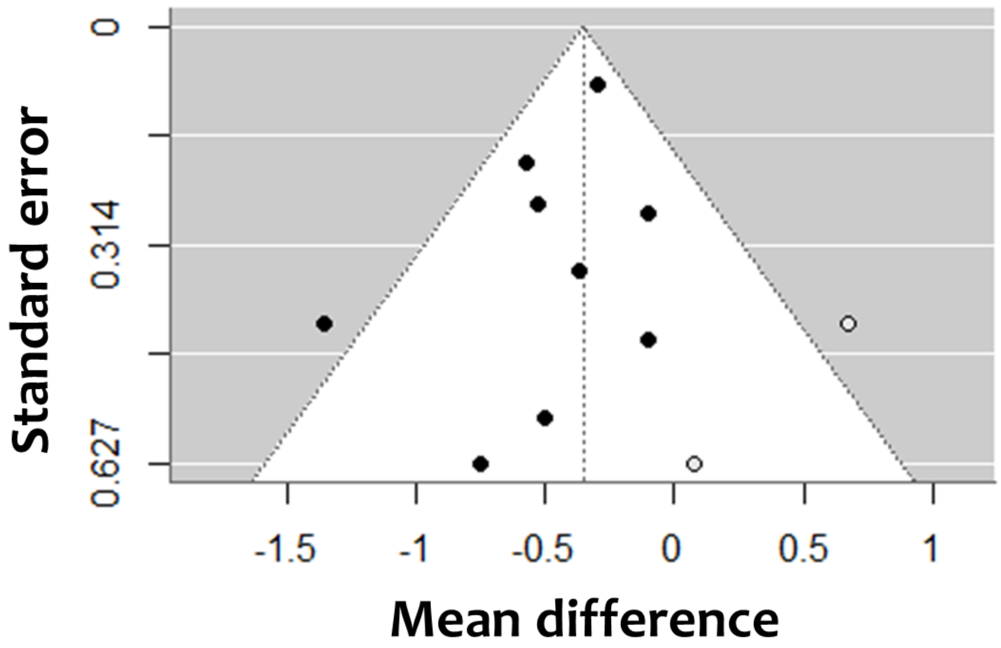

Suppl. Figure 13. Funnel plot of HbA1c before and after SGLT2-i therapy. Open circles represent missing studies.

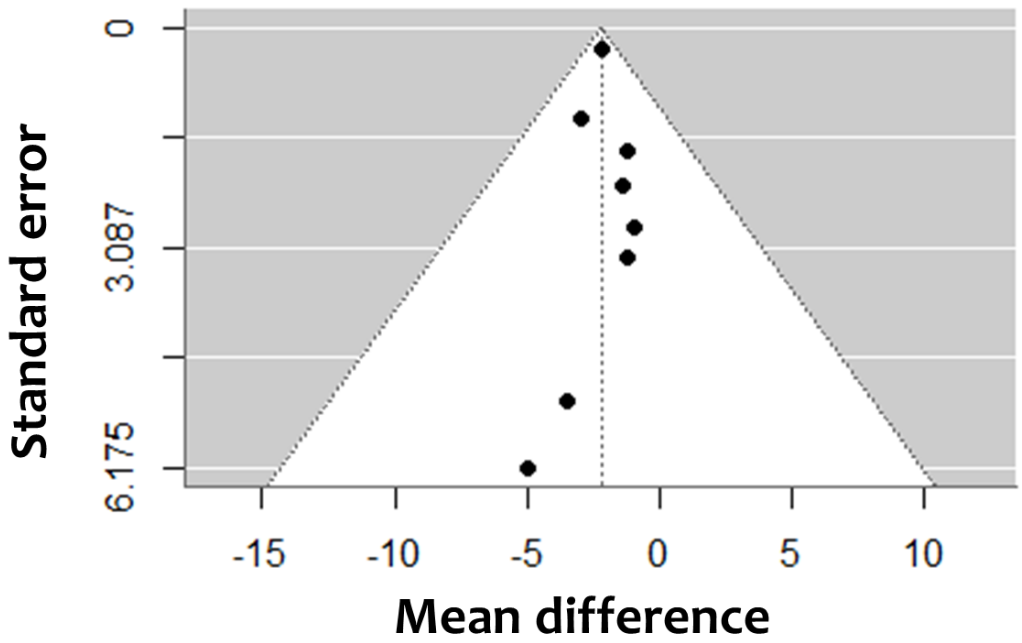

Suppl. Figure 14. Funnel plot of body weight before and after SGLT2-i therapy. Open circles represent missing studies.

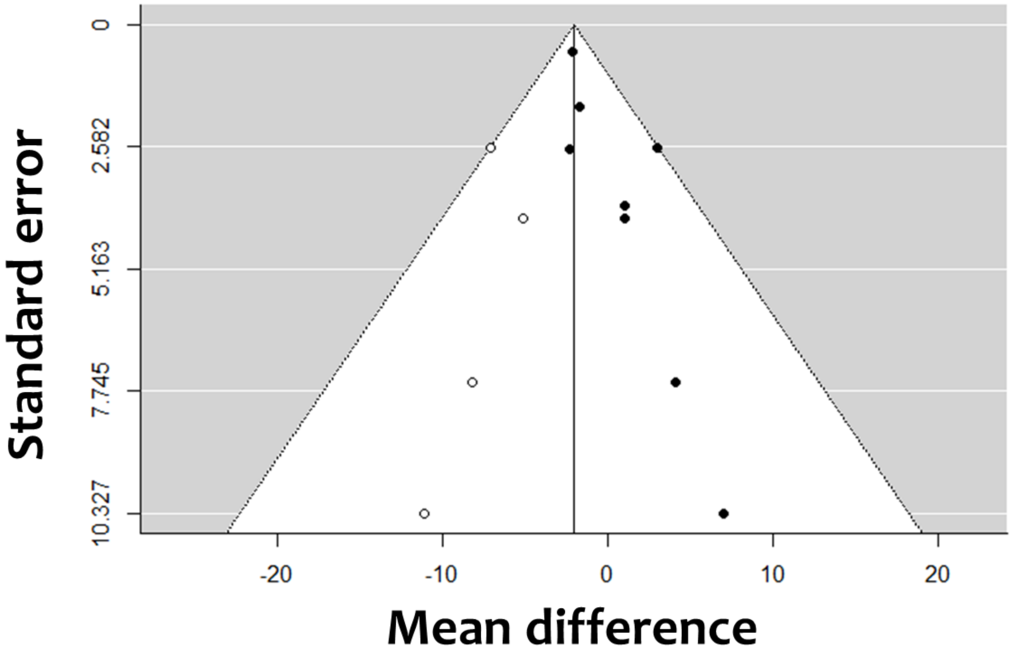

Suppl. Figure 15. Funnel plot of estimated glomerular filtration rate before and after SGLT2-i therapy. Open circles represent missing studies.

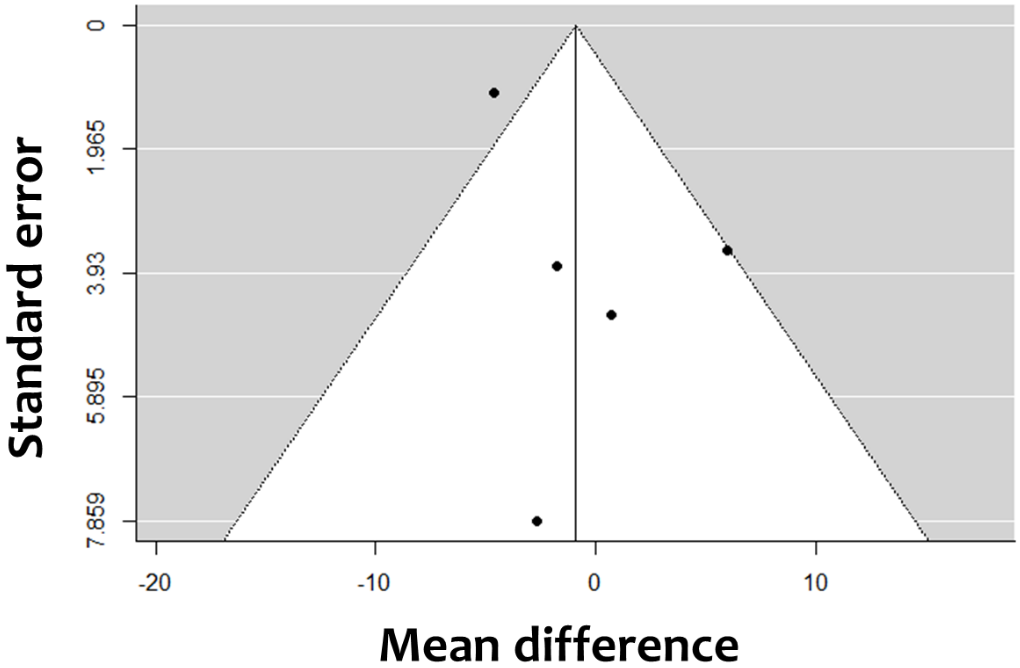

Suppl. Figure 16. Funnel plot of systolic blood pressure before and after SGLT2-i therapy. Open circles represent missing studies.
